# Outer-Sphere Hydrogen Bonds Amplify Mutual Actinide Separation

**DOI:** 10.1021/acscentsci.6c01133

**Published:** 2026-09-18

**Authors:** Bin Li, Zhiyuan Zeng, Ziyi Zhang, Yu Kang, Zehui Xu, Xiaoyan Tang, Li Wang, Chao Xu

**Affiliations:** a Institute of Nuclear and New Energy Technology, 12442 Tsinghua University, Haidian District, Beijing 100084, China; b Department of Chemistry, 12379 Capital Normal University, Haidian District, Beijing 100048 China; c Beijing National Laboratory for Molecular Sciences, Key Laboratory of Polymer Chemistry and Physics of Ministry of Education, Center for Soft Matter Science and Engineering, College of Chemistry and Molecular Engineering, 12465 Peking University, Haidian District, Beijing 100871, China

## Abstract

Mutual actinide separation is critical for nuclear waste remediation, superheavy-element production, and f-block-element-based radiopharmaceuticals, yet it remains a formidable challenge owing to the high chemical similarity of these elements and the harsh conditions typically encountered, such as high acidity and intense radioactivity. Here, we show that combining lipophilic **TODGA** with the histidine-decorated hydrophilic ligand **Phen-2DIHis** enables the efficient separation of Am­(III), Cm­(III), and Cf­(III) under highly acidic conditions. This system provides substantially enhanced separation factors for both Am­(III)/Cf­(III) (383) and Cm­(III)/Cf­(III) (36.9) compared to benchmark methods while maintaining practical metal distributions by directing the targeted actinide ions into opposite phases. Spectroscopic titrations and slope analyses support predominant 1:1 metal–ligand complexation. Theoretical calculations further suggest that nitrate-involved outer-sphere hydrogen bonding from the histidine termini may contribute unexpectedly to the thermodynamic bias for separation processes. These results highlight weak interactions beyond the inner coordination sphere as a critical design handle for mutual actinide separation under strongly acidic conditions.

## Graphical abstract

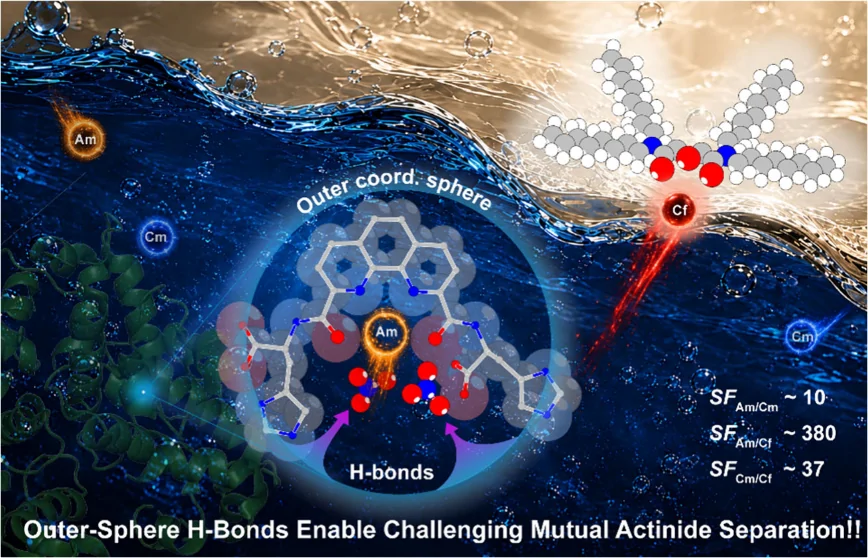

## 1 Introduction

Actinide separation remains one of the most challenging tasks in radiochemistry as actinide lies at the intersection of fundamental f-block element chemistry, isotope production, nuclear technology, and radiological safety.^1–5^ For actinides beyond plutonium (Pu), americium (Am), curium (Cm), and californium (Cf) are especially important. Am isotopes are used in industrial gauges and smoke detectors and are also considered for radioisotope power.^6–8^ Cm isotopes are candidate power sources for deep-space power systems.^7,9^ Am and Cm serve as feedstock elements for other heavy isotope production, especially for Cf (Figure 1), which is the heaviest actinide available in quantities sufficient for macroscopic chemical studies and provides an intense spontaneous-fission neutron source for reactor start-up, neutron radiography, activation analysis, well logging, and neutron therapy applications.^2,3,10–13^ Actinide separation and purification serve as prerequisites for high-purity isotope production, understanding the periodic trends, and subsequent nuclear applications.^1,14–18^ Yet Am, Cm, and Cf typically coexist during the fission process in the strongly acidic aqueous medium. Chemically, they are dominated with trivalent states, possess closely related ionic radii, and display only subtle differences in metal–ligand bonding.^1,3,19–21^ Efficient separation methodologies are currently highly pursued to separate especially for Am/Cf and Cm/Cf to, on one side, guarantee the supply of the heavy radioactive actinide such as Cf while, on the other side, shed light on precise design of chemical engineering separation process for f-block elements.^1,19^

**Figure 1:**
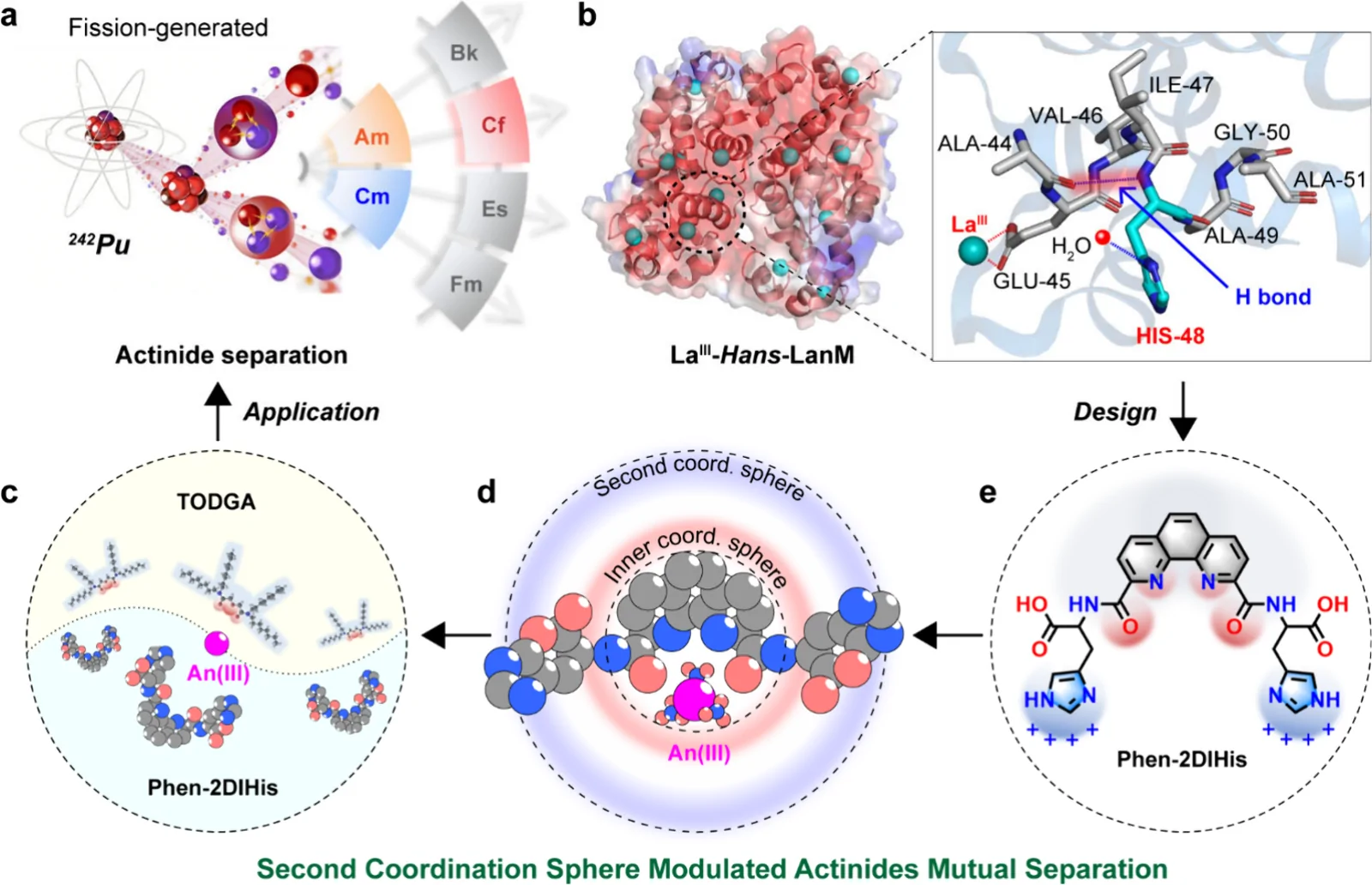
Ligand design. (a) Schematic illustration of the fission-based production pathway from ^242^Pu to Am, Cm and Cf. (b) Structure of the La^III^-*Hans*-LanM complex (left, PDB Entry: 8DQ2 (10.2210/pdb8dq2/pdb) and the zoomed-in metal coordination sites (right, EF1 domain). Local hydrogen bond (H-bond) was emphasized in red. The highlighted local hydrogen-bonding contacts exemplify a second-coordination-sphere control mechanism in which noncovalent interactions outside the direct metal–ligand coordination sphere help organize the metal-binding environment and transmit subtle metal-size differences into distinct coordination stability. (c) Illustration of the biphasic “tug-of-war” extraction system where **TODGA** is the lipophilic extractant used in the organic phase and **Phen-2DIHis** serves as the aqueous masking ligand. (d) Schematic depiction of the coordination environment around the inner coordination sphere (red shaded region, colored by element: gray = C, blue = N, red = O, pink = An­(III)) and the second coordination sphere containing the non-coordinating terminal groups and associated noncovalent interactions (blue shaded region). (e) Chemical structure of the **Phen-2DIHis**.

Despite decades of progress, Cf separation from Am and Cm remains a formidable challenge.^1,3,14,22^ Current Cf purification relies heavily on chromatographic flowsheets developed for isotope-production campaigns.^2,11,23^ High-chloride anion-exchange and α-hydroxyisobutyric acid-based cation-exchange elution have been used to separate Am/Cm from other heavy fractions and to purify Bk/Cf/Es/Fm products from one another. These workflows are robust while still require multistep column operations, stringent control of feed composition and elution conditions, long processing times and repeated recycle streams which lead to material hold-up and decay loss. Solvent extraction offers an attractive alternative because it is amenable to stagewise operation, readily scalable, compatible with continuous operation and well suited to highly acidic process media.^24,25^ However, reported solvent extraction systems for Am/Cm/Cf separation remain limited.^14–18,23,26–30^ Industrial-friendly ligands such as **TODGA** can recover trivalent actinides (An­(III)) and lanthanides (Ln­(III)) efficiently from acidic feeds but displays limited efficiency for adjacent An­(III).^31^ Other extractants and/or multicomponent systems examined for Am/Cm, Am/Cf or Cm/Cf separations which include organophosphorus reagents,^7,32,33^ N,O-donor amides,^7,14^ **BTBP**-type ligands and related soft-donor systems^7,34–36^ but generally give unsatisfactory separation factors (typically smaller than 10, Tables S1 and S2). Typically, the reported low separation efficiencies originated from the following points: 1) similar to trivalent lanthanide/actinide separation in nuclear waste remediation, mutual actinide separation was conducted in strong acid where acid–base interactions seriously interfered with metal coordination especially for polypyridyl ligands;^24,37^ 2) most actinides were highly radioactive and limited solution coordination data were available;^1,22^ 3) the almost identical electronic structures especially for adjacent actinides invalidate the general coordinating/separating ligand design role of hard–soft acid–base (HSAB);^7^ 4) recent reports indicated the knowledge in lanthanide separation might not be directly transferred to actinide separation;^38,39^ and 5) current reported Am–Cm–Cf separation methods suffered from undesired metal distributions which may not be practically adaptable.^7,14^

Inspired by recent development in aqueous masking ligands for f-block element separation, we believe the biphasic “tug-of-war” strategy could provide a promising alternative approach to address the above challenges (Figure 1). In such systems, a less-selective, industrial friendly extracting lipophilic ligand in the organic phase competes with a selective hydrophilic masking ligand in the aqueous phase. Metal ions that are more strongly bound by the hydrophilic ligand would remain in the aqueous phase whereas less strongly masked ions are transferred to the organic phase by the extractant. Such strategy can, in principle, amplify small thermodynamic differences of f-block elements by combination of two extractants with the overall separation efficiency relying highly on the rational design of hydrophilic ligands.^24,37,40,41^ Currently, most of the literature work focuses on the chemical modification of the binding pocket of the ligand (inner-sphere).^24,42–44^ Factors such as preorganization, ligand electronic effects and adaptation of ligand asymmetry were investigated to fine tune the selectivity which were chemically sophisticated and time-consuming based on trial-and-error processes.^24,43^ As promising new frontiers for f-block elements discrimination and separation, ligands/materials with balanced inner-sphere coordination and second coordination sphere interactions to amplify the traditional radius-based f-block element discrimination were receiving increasing attention while only limited systems were reported so far.^45–49^ Here, the second coordination sphere refers to the noncovalent interaction environment beyond the donor atoms directly coordinated to the metal center, including hydrogen bonding, electrostatic interactions, solvent organization, and conformational effects.

In the current manuscript, we show that a histidine-decorated phenanthroline-based hydrophilic ligand, **Phen-2DIHis**, which combines a preorganized N,O-donor core with proton-responsive histidine termini (Figure 1), delivers order-of-magnitude improvements in Am/Cf and Cm/Cf separation relative to typical solvent-extraction processes. UV–vis spectroscopic titrations, NMR titrations, and slope analyses support predominant 1:1 metal–ligand complexation, while DFT calculations further suggest that nitrate-involved outer-sphere hydrogen bonds between the histidine termini and coordinated nitrates around An­(III) contribute to the differentiated stabilization of Am­(III), Cm­(III), and Cf­(III) complexes thereby leading to superior actinide separation performance. Nitrate groups are typically regarded as charge-balancing anions or coordinating groups that satisfy the high coordination numbers of lanthanides and actinides.^50–53^ In the present system, however, we show that nitrate-involved outer-sphere hydrogen bonds can serve as an underappreciated design element for amplifying subtle thermodynamic differences among neighboring trivalent actinides in solvent extraction systems. Accordingly, nitrate-involved outer-sphere hydrogen bonding is considered to be a specific mechanism through which second-coordination-sphere modulation is achieved. More broadly, this work demonstrates that noncore terminal-group engineering can tune the second coordination sphere, providing an effective strategy for separating similar metal cations under highly acidic conditions.

## 2 Results and Discussion

### 2.1 Evaluation of (Am, Cm)/Cf Separation Performance

The extraction and separation performances of **Phen-2DIHis** for Am­(III), Cm­(III), and Cf­(III) were evaluated in a biphasic system composed of **TODGA** in the organic phase and the hydrophilic ligand **Phen-2DIHis** in the aqueous phase. Parameters such as metal distribution (*D*_M_) and separation factor (*SF*_An/An_) (Supporting Information Note 1) were used to quantitatively show the ligand separation capability. As depicted in Figure 2a, optimized Am/Cm, Am/Cf and Cm/Cf separations factors of 2.4 (corresponding to 2.0 M HNO_3_), 14.3 (corresponding to 0.5 M HNO_3_) and 9.3 (corresponding to 0.5 M HNO_3_) were observed with general increasing *D*_M_ at higher acid concentrations. These results were consistent with literature reports showing the weak mutual discrimination ability of **TODGA**, but the similar *D*_M_ could be a major concern as both metal cations tend to preferentially migrate into the same phase.^54^

**Figure 2:**
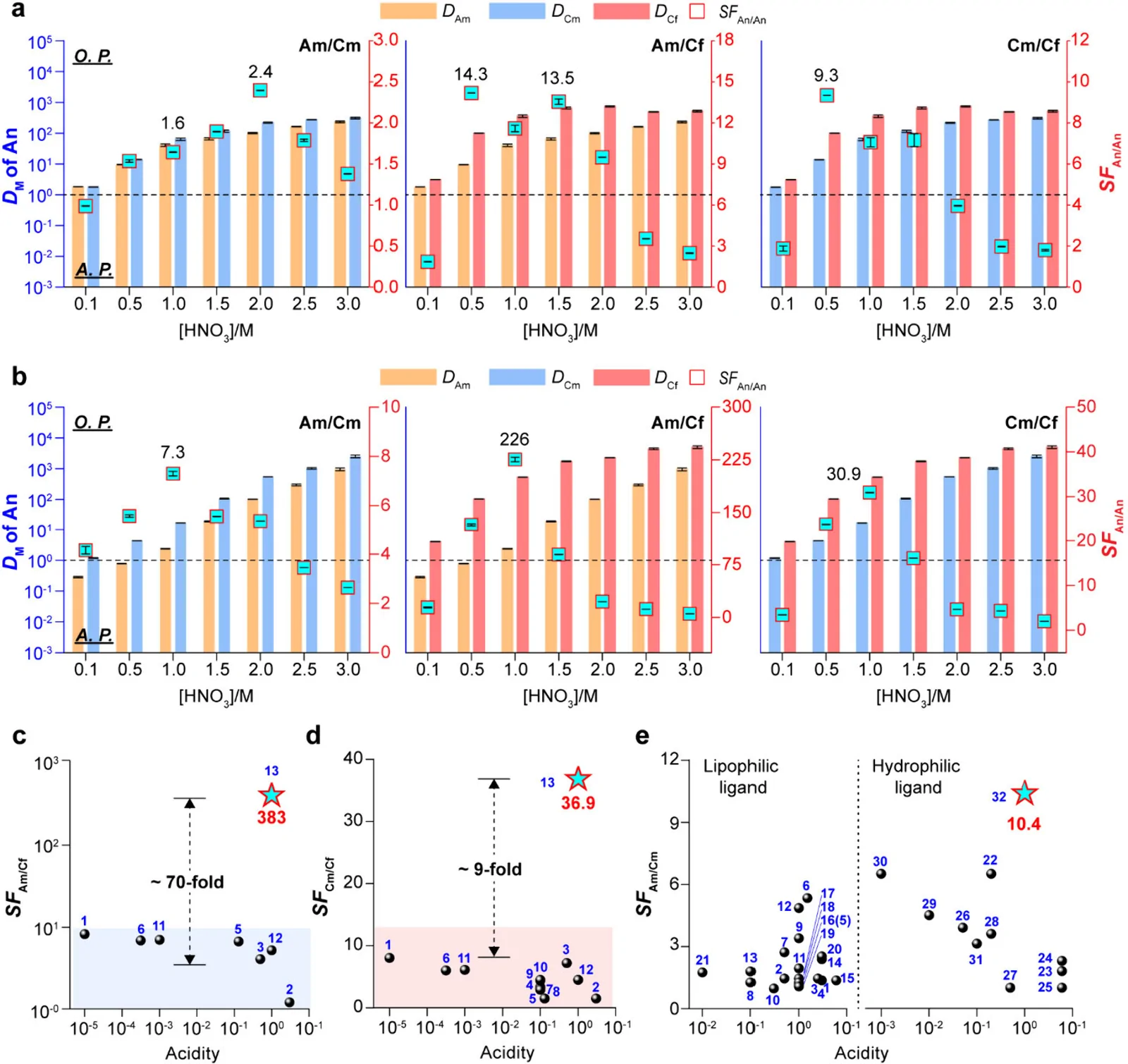
Separation performance for Am­(III), Cm­(III) and Cf­(III). Extraction performances for Am­(III), Cm­(III) and Cf­(III) as functions of HNO_3_ concentrations for **TODGA** without (a) and with (b) aqueous masking **Phen-2DIHis** ligand. Comparison of the best of *SF*_Am/Cf_ (c), *SF*_Cm/Cf_ (d) and *SF*_Am/Cm_ (e) in this work (blue stars) with representative literature values (black circles). The numbers in panels c and d correspond to the entries listed in the ‘No.’ column of Table S1, whereas those in panel e correspond to the entries in Table S2. Experimental conditions: 0.1 M **TODGA** in *n*-dodecane/*n*-octanol (90/10, v/v) as the organic phase, O; the aqueous phase consisted of HNO_3_, with or without 10 mM **Phen-2DIHis**; *T* = 298 K and O/A = 1:1 for 120 min. For experimental conditions in panel (c–e), Supporting Information Tables S1 and S2. Data were presented as mean ± standard deviation from three independent extraction experiments (*n* = 3); error bars represented standard deviation.

The separation behavior changed significantly after **Phen-2DIHis** was introduced into the aqueous phase. Across the examined acidity range, **Phen-2DIHis** suppressed the extraction of all three metal ions to different extents (Figure 2b). This masking effect followed the order Am­(III) > Cm­(III) > Cf­(III) and was especially pronounced at low to intermediate acidities (0.1–2.0 M). Specifically, at 0.1 M HNO_3_, *D*_Am_ decreased to 0.28 from 4.60 with only **TODGA** indicating most Am­(III) was retained in the aqueous phase whereas Cm­(III) distributed in both phases (*D*_Cm_ = 1.18) and Cf­(III) was preferentially extracted to the organic phase (*D*_Cf_ = 4.08). This led to the increasing separation for Am­(III), Cm­(III), and Cf­(III). Systematic optimization of the extraction conditions (acidity, ligand concentration, NaNO_3_ and contacting times, Figure S1) led to the best *SF*_Am/Cf_ and *SF*_Cm/Cf_ of 383 and 36.9, respectively. To the best of our knowledge, these values are among the highest reported to date for the mutual separation of trivalent Am­(III), Cm­(III), and Cf­(III) by liquid–liquid extraction.^7,14–18,23,26–28,55–57^ Although only limited separation data were available in the literature for Cf­(III) because of its scarcity, the current reported data displayed about 70- and 9-fold separation enhancement with more favorable Am­(III)/Cm­(III) and Cf­(III) distributions (Table S1, with Am­(III)/Cm­(III) in aqueous phase while Cf­(III) was selectively extracted by **TODGA** in current work).

To have a better idea of how the current system perform, the mutual separation of Am­(III)/Cm­(III) were also investigated and the results were given in Figure 2e. Cm­(III) isotopes were short-lived, intensely radioactive, and strong neutron emitters, which were proposed to be separated as early in the separation process as possible in order to develop a compact separation process.^7^ Besides, Cm­(III) serves as a key target for Cf­(III) production, and Am­(III) is among the transmutation targets considered for replenishing the Cm supply.^10,11^ Furthermore, more available data can be found in the reported work for Am­(III)/Cm­(III) separation. Under the optimized conditions, an *SF*_Am/Cm_ of 10.4 could be deduced with *D*_Am_ and *D*_Cm_ values of 0.75 and 7.78. These data further confirmed that the **Phen-2DIHis/TODGA** system combined high selectivity with a practically favorable operating window in terms of metal distributions, separation factors, solution acidity, ligand loading, and contact time.

### 2.2 Identification of Solution Coordination Species by NMR Titrations

To clarify the coordination origin of the observed Am­(III)/Cm­(III)/Cf­(III) selectivity, as discussed in the previous section, we investigated the solution coordination behavior of **Phen-2DIHis** by ^1^H NMR titrations. As direct spectroscopic studies of Am­(III)/Cm­(III)/Cf­(III) are limited by their scarcity, tracer-level availability, and radiological hazards, Nd­(III)/Sm­(III)/Gd­(III) was employed as an experimentally accessible lanthanide surrogate spanning a relevant trivalent ionic-size range.^38,39^ These lanthanides were used as practical and structurally informative models rather than as strict equivalents of the corresponding actinides. Also, these models are appropriate for examining the gross coordination mode and size-dependent response because late An­(III) chemistry is often predominantly ionic and lanthanide-like.^5,38^ Nevertheless, ligand-dependent 4f/5f bonding differences preclude their use as strict quantitative substitutes for Am­(III), Cm­(III), and Cf­(III). The titrations were performed in 1.0 M DNO_3_/D_2_O with 25 mM **Phen-2DIHis** to resemble the acidic aqueous conditions used in the actinide-extraction experiments.

Upon addition of Nd­(III), the phenanthroline aromatic protons of **Phen-2DIHis** (protons 1–3, blue region in Figure 3b; full spectra in Figure S2) showed pronounced attenuation and downfield shifts indicating coordination at the neighboring N,O-donor sites. In contrast, the imidazole-related protons from the histidine termini (protons 4 and 5, gray region in Figure 3b) remained nearly unchanged suggesting that the histidine groups do not directly participate in inner-sphere coordination. When the M/L ratio exceeded about 0.3, a second set of downfield-shifted resonances appeared and gradually increased in intensity. Even at 5 equiv of Nd­(III), only two sets of resonances were observed, which can be assigned to the free ligand and a 1:1 Nd­(III)­L species with no evidence for higher-stoichiometry complexes. Similar spectral evolution was observed for Sm­(III), including downfield shifts of protons 1–3 and the emergence of new peaks in the 7.8–8.4 ppm region (Figure 3c and Figure S3), supporting formation of a 1:1 Sm­(III)­L species. For Gd­(III), the ligand signals shifted and broadened at low metal loading probably because of the strong paramagnetism of Gd­(III) (Figure 3d and Figure S4). Nevertheless, considering the relatively smaller radius of Gd­(III) with respect to Nd­(III) and Sm­(III), we expected the consistent predominant of Gd­(III)­L formation as the smaller metal centers could not physically accommodate more ligands in the case of Gd­(III), which was further confirmed by absorption titration in the following discussion. Overall, these NMR titration results illustrate that **Phen-2DIHis** predominantly forms 1:1 M/L complexes under the present extraction conditions.

**Figure 3:**
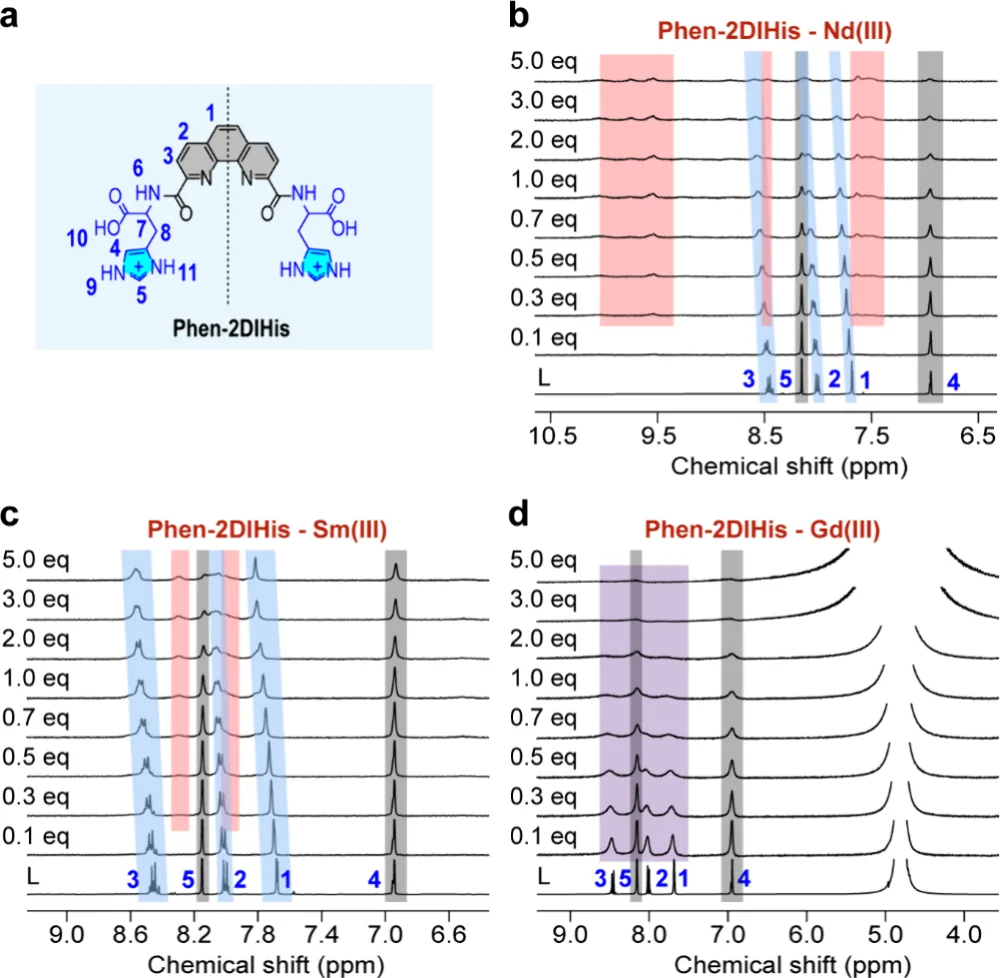
NMR titrations of **Phen-2DIHis** with Nd­(III), Sm­(III) and Gd­(III). (a) Ligand structure of **Phen-2DIHis** with the NMR active protons assigned and numbered. Stacked, zoomed-in ^1^H NMR spectra of **Phen-2DIHis** titrated with Nd­(NO_3_)_3_ (b), Sm­(NO_3_)_3_ (c), and Gd­(NO_3_)_3_ (d). Metal/ligand ratios were given at the left of each panel. Shaded regions highlighted resonances assigned to the free ligand and the 1:1 metal–ligand species (blue and gray for ligand, red for the M/L = 1:1 complex of Nd­(III) and Sm­(III), while purple for the M/L = 1:1 complex of Gd­(III)). Spectra were recorded for 25 mM **Phen-2DIHis** in 1.0 M DNO_3_/D_2_O titrated with 0.1 M Nd­(NO_3_)_3_, Sm­(NO_3_)_3_ or Gd­(NO_3_)_3_ at 25 °C.

### 2.3 Coordination Modes and Thermodynamic Analyses

UV–vis absorption titrations were performed to further confirm the coordination stoichiometry and obtain quantitative formation constants for interpreting the extraction performance (Figure 4 and Figure S5). Upon addition of Nd­(III), **Phen-2DIHis** displayed a clear red shift of the absorption band from 287 to 296 nm, accompanied by increased absorbance intensity, which confirms effective metal–ligand coordination. *HypSpec* fitting indicated the predominant formation of ML species at 1. The titrations with Sm­(III) and Gd­(III) showed similar spectral evolution, supporting the same coordination mode across this lanthanide series. Notably, the evolution of the ML species followed the order Nd­(III)­L > Sm­(III)­L > Gd­(III)­L, as reflected by the faster growth of the 1:1 species, higher final ML fractions, and larger stability constants. At the end of the titrations, the calculated fractions of Nd­(III)­L, Sm­(III)­L, and Gd­(III)­L reached 49.6%, 39.7%, and 36.0%, respectively. This affinity trend of Nd­(III) > Sm­(III) > Gd­(III) follows the decreasing ionic radius sequence used to model Am­(III), Cm­(III), and Cf­(III), providing a qualitative, size-dependent thermodynamic reference for the extraction selectivity.

**Figure 4:**
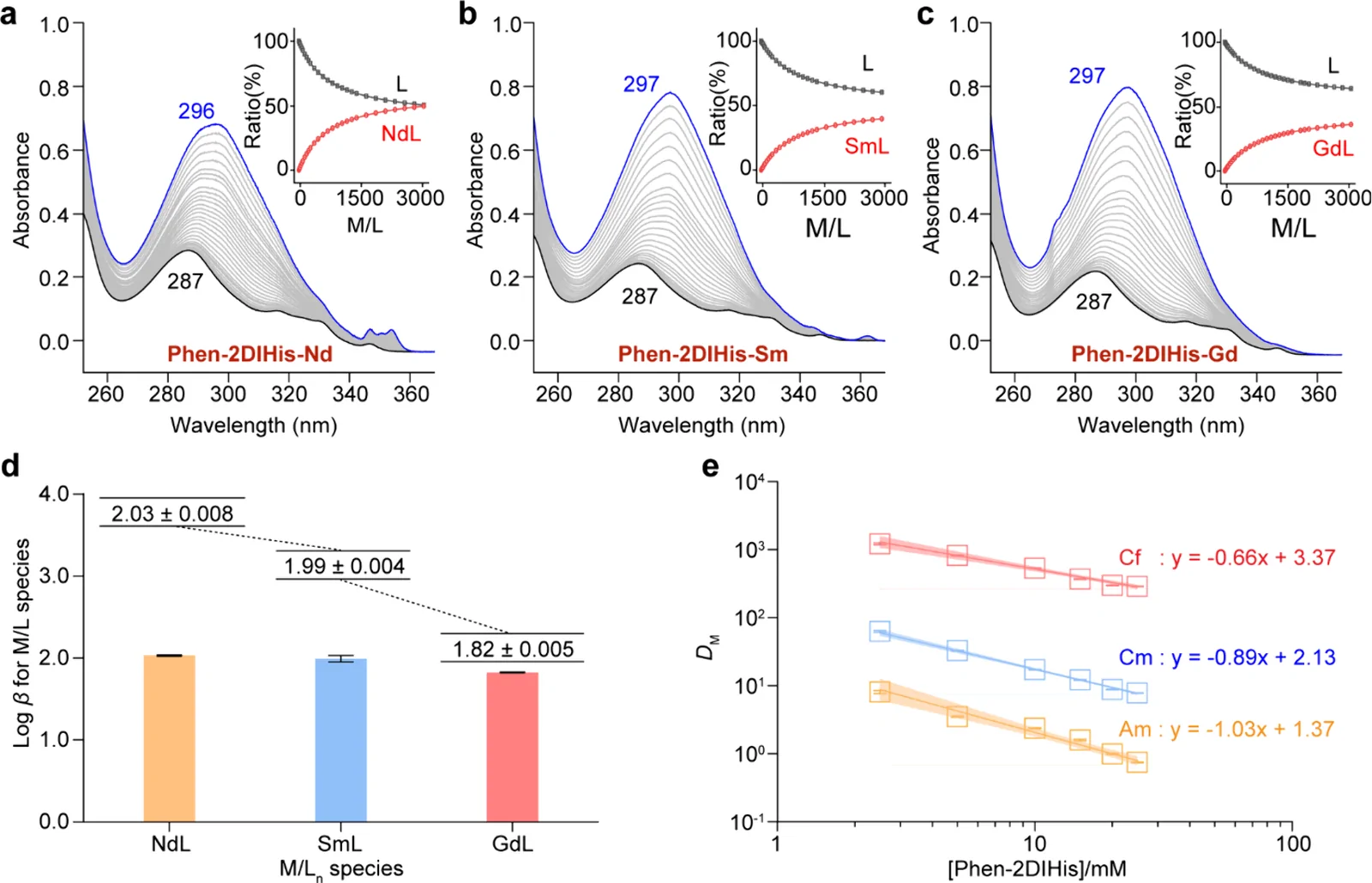
Coordination thermodynamic analyses of **Phen-2DIHis** with Nd­(III)/Sm­(III)/Gd­(III). UV–vis absorption titrations of **Phen-2DIHis** with Nd­(NO_3_)_3_ (a), Sm­(NO_3_)_3_ (b), and Gd­(NO_3_)_3_ (c). The black and blue curves represent the ligand solution absorption spectra before and after titrations. Inset figures show the fitted species fractions of all species derived from absorption titrations. (d) Formation stability constants (log *β*) for ML species obtained from absorption titration data. Experimental conditions: Ligand and metal concentrations were 0.01 and 16 mM with titration conducted in 0.01 M HNO_3_ with ionic strength controlled by 0.1 M NaNO_3_. (e) Am­(III) (golden), Cm­(III) (blue) and Cf­(III) (red) distribution ratios of as functions of ligand concentrations. Experimental conditions: 0.1 M **TODGA** in *n*-dodecane/*n*-octanol (90/10, v/v) as the organic phase, O; the aqueous phase consisted of 1.0 M HNO_3_ with **Phen-2DIHis** concentrations in the range of 5 mM to 25 mM; *T* = 298 K and O/A = 1:1 for 120 min. Data were presented as mean ± standard deviation from three independent extraction experiments (*n* = 3); error bars represented standard deviation.

To verify whether the same stoichiometry was retained under biphasic extraction conditions, ligand-concentration-dependent metal distribution experiments were conducted for Am­(III), Cm­(III), and Cf­(III) at a fixed acidity (Figure 4e). Linear fitting of log *D*_M_ versus log [**Phen-2DIHis**] gave slopes of −1.03, −0.89, and −0.66 for Am­(III), Cm­(III), and Cf­(III), respectively. The absolute values, close to or below unity, exclude dominant formation of 1:2 metal-to-ligand species and instead support predominant 1:1 M/L complexation for all three actinides.^58^ Moreover, the slope magnitudes decrease in the order Am­(III) > Cm­(III) > Cf­(III), consistent with the UV–vis-derived binding trend and the observed extraction sequence. Overall, the NMR titrations, UV–vis titrations, and extraction slope analyses consistently support predominant 1:1 M/L complexation with binding affinities following the same trend as the extraction performance.

### 2.4 Computational Elucidation of Outer-Sphere H-Bond within the Second Coordination Sphere in Actinide Selectivity

In the previous sections, we have demonstrated that the current reported biphasic system composed of **TODGA** and **Phen-2DIHis** displayed superior Am­(III)/Cm­(III)/Cf­(III) separation capability with the predominant coordination species of M/L = 1. In the following discussions, complex-level driving forces were unrevealed through calculation methods because of the low crystallinity of both the ligand and the complexes preventing the single-crystal cultivations and analyses.

On the basis of our previous reports on similar ligand architectures,^37,40,59^ all the calculations for **Phen-2DIHis**/An­(III) were based on the tetradentate **Phen-2DIHis** with O,N,N,O binding sites and the 10-coordinated metal centers. Under such circumstances, three possible complex architectures existed concerning the orientation of the chiral carbon adjacent to the imine groups (position 7 in Figure 3). As exemplified in the case of Am­(III) (Supporting Information Note 2, Figure S6), molecular structure optimization based on density functional theory (DFT) calculations was conducted and the geometry with one of the carboxylic groups pointing to the metal center was proved to be the most stable structure (Figure S6). Successively, the free energy changes of the extraction processes were divided into two elemental steps with 1) the formation of [**Phen-2DIHis**]­[An­(NO_3_)_3_] complexes and 2) the formation of [**TEDGA**]_3_[An­(III)]­(NO_3_)_3_ where **TEDGA** was used to represent **TODGA** concerning both the time cost for calculations and the ligand water solubilities, as it retains the same tridentate O,O,O donor core while replacing the peripheral *n*-octyl groups with ethyl groups. As the same model was applied to Am­(III), Cm­(III), and Cf­(III), the calculated energies were used to compare their relative trends within an identical local coordination framework rather than the absolute extraction thermodynamics of **TODGA**. As depicted in Figure 5a and Tables S3 and S4, the free energy changes for [**Phen-2DIHis**]­[An­(NO_3_)_3_] formation follow the order of Am­(III) > Cm­(III) > Cf­(III) (Table S3) while the conversion free energy changes for [**TEDGA**]_3_[An­(III)]­(NO_3_)_3_ displayed the reverse trend of Am­(III) < Cm­(III) < Cf­(III) (Table S4). Specifically, as sufficiently reliable solvent-model parameters and dispersion-correction data for Am, Cm, and Cf were unavailable within the present Gaussian-based computational framework, the three actinide systems were evaluated in the gas phase with the same ligand model, coordination stoichiometry, and computational protocol. The resulting energies were therefore used primarily to compare the relative energetic trends of the Am­(III), Cm­(III), and Cf­(III) systems under an internally consistent idealized condition rather than to reproduce quantitative solution-phase extraction free energies. Solvation, protonation equilibria, ionic strength effects, and explicit HNO_3_/H_2_O interactions may influence the absolute energies. Although these gas-phase energetic estimations were under ideal conditions without considering the solvent interactions, the calculation results well explained the observed extraction results as discussed previously.

**Figure 5:**
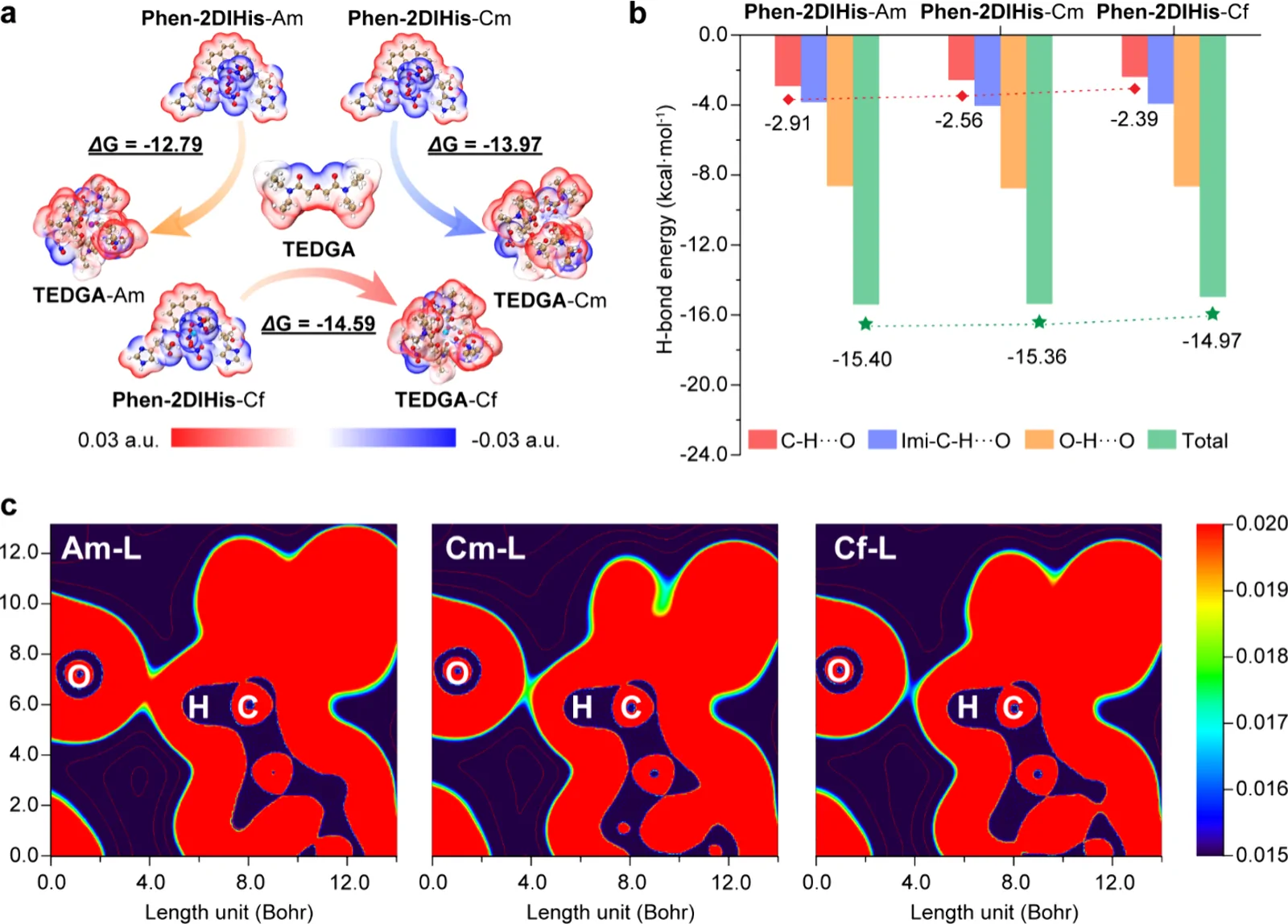
Driving force analyses for Am­(III), Cm­(III) and Cf­(III) separation. (a) Calculated free energy changes (Δ*G*) during extraction, **TEDGA** was used to replace **TODGA**. (b) Comparison of H-bond energies in **Phen-2DIHis/**An­(III) complexes. (c) Two-dimensional ELF maps for C-H···O hydrogen bond in **Phen-2DIHis/**An­(III) complexes.

Further bonding analyses were performed to gain atom-level insights into the driving forces underlying the separation. As summarized in Table S5, the optimized structures of the Am­(III), Cm­(III), and Cf­(III) complexes generally show shorter M–O bonds than M–N bonds consistent with the harder donor character of oxygen.^59^ In addition, both the M–O and M–N bond lengths decrease from Am­(III) to Cm­(III) and Cf­(III) suggesting stronger inner-sphere coordination for the smaller Cf­(III) ion. This trend follows the expected lanthanide/actinide contraction but appears inconsistent with the calculated formation free energies discussed above and with the experimentally observed extraction behavior. We therefore hypothesized that additional factors, beyond simple cation-size-driven inner-sphere coordination, may contribute oppositely and preferentially stabilize the complexes of the relatively larger actinide ions such as Am­(III) and Cm­(III).

Considering that the coordinated nitrate groups can participate in hydrogen bonding with the carboxylic O–H, imidazole C–H, and other active hydrogen bearing groups of **Phen-2DIHis**, we further analyzed the nitrate-involved outer-sphere hydrogen-bonding interactions within the second coordination sphere using the Independent Gradient Model based on Hirshfeld partition (IGMH) method. IGMH analysis was employed to visualize and compare the noncovalent interaction regions surrounding the actinide binding sites with particular focus on the nitrate-involved outer-sphere hydrogen-bonding network. As shown in Figure S7, three representative hydrogen-bonding contacts were identified around the metal centers: the O–H···O interaction between the carboxylic acid group and the nitrate group, denoted as O–H···O; the C–H···O interaction between the imidazole C–H and nitrate group, denoted as Imi-C–H···O; and the C–H···O interaction between the C5–H group (Figure 3) and nitrate group, denoted as C–H···O. Complementary electron localization function (ELF) analysis indicated that the O–H···O interaction is the strongest among the three hydrogen bonds and exhibits a moderate degree of covalent character (Figure S8). In contrast, the C–H···O and Imi-C–H···O contacts are weaker and are dominated by electrostatic interactions consistent with their low core-valence bifurcation (CVB) values (Table S6). Given that the extraction process requires **TODGA** to disrupt the [**Phen-2DIHis**]­[An­(NO_3_)_3_] complexes and form extractable lipophilic actinide species, we estimated the hydrogen-bond energies of the three representative interactions for each actinide complex (Supporting Information Note 3). The results are shown in Figure 5b. The overall hydrogen-bond energy trend is consistent with the calculated formation free energies of [**Phen-2DIHis**]­[An­(NO_3_)_3_] with the Am­(III) complex being the most energetically favored. Moreover, the calculated hydrogen-bond energy scale matches well with the Gibbs free energy changes associated with the extraction process (Figure 5a). These results suggest that the superior Am­(III)/Cm­(III)/Cf­(III) separation performance of the present system may primarily be governed by the nitrate-involved outer-sphere hydrogen-bonding network rather than by conventional cation-radius-controlled inner-sphere coordination thermodynamics. However, the quantitative magnitude of this contribution under the experimental extraction conditions cannot be determined from the present computational model alone.

### 2.5 Actinide Separation in Simulated Feedstocks

At last, the Am­(III)/Cm­(III)/Cf­(III) separation abilities of **Phen-2DIHis/TODGA** were further evaluated under practical feedstocks containing multiple competing lanthanides, fission products, and matrix ions that may influence metal distribution and phase-transfer behavior. To show the robustness of current methods, we therefore tested the **Phen-2DIHis/TODGA** system in two simulated complex feedstocks: an HFIR (High Flux Isotope Reactor)-relevant multi-lanthanide simulant and a simulated high-level liquid waste (HLLW) solution based on the **TRPO** process (compositions in Supporting Information Note 5 and Table S7). As shown in Figure 6, the characteristic selectivity pattern was retained in both media, although the separation factors decreased moderately compared with those in the simplified system. In the HFIR and HLLW feeds, the system afforded *SF*_Am/Cm_ values of 7.42 and 6.22, *SF*_Am/Cf_ values of 196 and 152, and *SF*_Cm/Cf_ values of 26.4 and 24.4, respectively. The reduced *SF*_An/An_ values mainly resulted from decreased Cf­(III) extraction with *D*_Cf_ decreasing from 287 to 161 and 149, whereas *D*_Am_ and *D*_Cm_ remained comparatively stable. The abundant trivalent lanthanides (especially La­(III), Ce­(III), Nd­(III), Eu­(III)) and Y­(III) present in both simulated feeds are the most likely primary contributors, as their co-extraction by **TODGA** can reduce the available organic-phase extractant capacity, to which Cf­(III) transfer is particularly sensitive. Additional HLLW components, including Pd, Ru, Rh, and Fe, may further contribute through ligand competition or matrix-dependent coordination effects, while the contribution of individual ions was not separately resolved as it was not the focus of the current research. Despite this matrix-induced decrease, the separation performance remained higher than that of the currently reported Cf­(III) extraction systems. These results demonstrate the robust anti-interference capability and superior mutual actinide separation performance of the present system under the application-relevant conditions.

**Figure 6:**
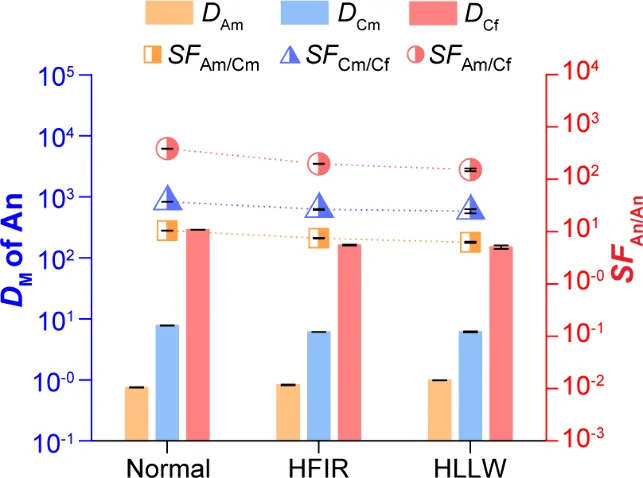
Separation performance in simulated feedstocks. Distribution ratios (*D*_M_) and separation factors (*SF*_An/An_) for Am­(III), Cm­(III) and Cf­(III) under different conditions. Normal refers to the aqueous phase with Am­(III), Cm­(III) and Cf­(III) in 1.0 M HNO_3_ while the compositions of HFIR and HLLW were given in Supporting Information. Experimental conditions: 0.1 M **TODGA** in *n*-dodecane/*n*-octanol (90/10, v/v) as the organic phase, O; the aqueous phase consisted of 1.0 M HNO_3_ with 25 mM **Phen-2DIHis**; *T* = 298 K and O/A = 1:1 for 120 min. Data were presented as mean ± standard deviation from three independent extraction experiments (*n* = 3); error bars represented standard deviation.

## 3 Conclusion

In this study, we developed an efficient biphasic “tug-of-war” system for the mutual separation of Am­(III), Cm­(III), and Cf­(III). By combining the lipophilic extractant **TODGA** with the histidine-decorated hydrophilic ligand **Phen-2DIHis**, the system achieved highly efficient actinide separation affording markedly enhanced separation factors for Am­(III)/Cf­(III) (383) and Cm­(III)/Cf­(III) (36.9) compared with current benchmark methods. Importantly, the system enabled favorable phase partitioning with Am­(III) and Cm­(III) preferentially retained in the aqueous phase while Cf­(III) was selectively transferred to the organic phase providing a practical operating window for separation. The robustness of this strategy was further demonstrated in HFIR- and HLLW-simulated feedstocks where high selectivity was retained despite the presence of multiple competing ions. NMR titrations, UV–vis spectroscopic titrations, and extraction slope analyses consistently supported predominant 1:1 complexation between **Phen-2DIHis** and the relevant trivalent metal ions. DFT calculations, together with IGMH and ELF analyses, further suggested that the histidine termini may contribute unexpectedly to selectivity by organizing nitrate-involved outer-sphere hydrogen-bonding interactions. These interactions introduce a thermodynamic bias across Am­(III), Cm­(III), and Cf­(III) counteracting the trend expected from simple cation-radius-controlled inner-sphere coordination. Overall, this work demonstrates that non-core terminal-group engineering can tune the second coordination sphere and amplify subtle differences among highly similar actinides providing both an effective separation strategy under highly acidic conditions and a new ligand-design principle for f-block nuclide separation.

## Supplementary Material



## Notes

Methods, characterization methods, solvent extractions, UV–vis and NMR titrations, DFT calculations, tables comparing literature data, Supporting Information Notes; figures of experimental conditions, NMR and absorption spectra, configurations, ELF color map; tables of binding energies, Gibbs free energies, bond lengths, electron densities, component list (PDF)
