# Supplementary material for: Outer-Sphere Hydrogen Bonds Amplify Mutual Actinide Separation
Source: ACS Cent Sci. 2026 Sep 18;12(10):1637–46. doi: 10.1021/acscentsci.6c01133 (PMC13720919; doi:10.1021/acscentsci.6c01133)
Supplement: Supplementary file 1 [file oc6c01133_si_001.pdf]

Supporting Information

Outer-Sphere Hydrogen-Bonds Amplify Mutual Actinide Separation

Bin Li,[a, ¶] Zhiyuan Zeng,[a, ¶] Ziyi Zhang,[b, c, ¶] Yu Kang,[a] Zehui Xu,[b] Xiaoyan Tang,[c] Li Wang[b, *] and Chao Xu[a, *]

[a] Institute of Nuclear and New Energy Technology, Tsinghua University, Haidian District, Beijing, 100084, China;

[b] Department of Chemistry, Capital Normal University, Haidian District, Beijing, 100048 China;

[c] Beijing National Laboratory for Molecular Sciences, Key Laboratory of Polymer Chemistry and Physics of Ministry of Education, Center for Soft Matter Science and Engineering, College of Chemistry and Molecular Engineering, Peking University, Haidian District, Beijing, 100871, China;

¶Bin Li, Zhiyuan Zeng and Ziyi Zhang contributed equally to this work.

* Corresponding authors: liwang862011@gmail.com (Li Wang); xuchao@tsinghua.edu.cn; (Chao Xu)

Contents

Materials……………………………………………………………………………………S3

Characterization Methods…………………………………………………………………S3

Solvent Extractions……………………………………………………………………S3

UV-vis Absorption Spectroscopy Titrations……………………………………………S5

Nuclear Magnetic Resonance Spectroscopy (NMR) Titrations…………………………S5

DFT Calculations……………………………………………………………………S5

Table S1 Comparison of representative literature reported Am(III)/Cf(III) and Cm(III)/Cf(III) separation performances with the current report……………………………………S7

Table S2 Comparison of representative literature reported Am(III)/Cm(III) separation performances by liquid-based extraction…………………………………………………S8

Table S3. Binding energies of Phen-2DIHis with Am(Ⅲ), Cm(Ⅲ), and Cf(Ⅲ) in nitric acid system……………………………………………………………………S18

Table S4. The Gibbs free energy change (*Δ*G) value of the competitive extraction reaction in this work…………………………………………………………………………S18

Table S5. The calculated bond lengths for Am(III), Cm(III), and Cf(III) with Phen-2DIHis at B3LYP/RECP/6-31G(d) level. ……………………………………………………S19

Table S6. The electron density value of the intramolecular hydrogen bond BCP in the complex molecule………………………………………………………………………S22

Table S7. Component list of the liquid material HLLW (with burnup of 33,000 MWd/tU, Am component in the TRPO process flow) ………………………………………………S23

Supporting Information Note 1……………………………………………………S10

Supporting Information Note 2……………………………………………………S16

Supporting Information Note 3……………………………………………………S16

Supporting Information Note 4……………………………………………………S22

Supporting Information Note 5……………………………………………………S23

Figure S1-S5…………………………………………………………………………S11

Figure S6……………………………………………………………………………S16

Figure S7-S8…………………………………………………………………………S20

SI references…………………………………………………………………………S24

Materials

All chemicals, reagents and solvents were obtained from commercial sources and used as received. Ultradry solvents were purchased from Energy Chemical Inc. and common salts or solvents (analytical or chromatographic grade) were sourced from Beijing TongGuang Fine Chemicals Co. **TODGA** was obtained from Qingdao Beitwall Technology Co., Ltd. Water (Milli-Q, 18.2 MΩ·cm). Neodymium(III), samarium(III) and gadolinium(III) nitrate hexahydrates ($Nd(NO_3)_3 \cdot 6H_2O$, $Sm(NO_3)_3 \cdot 6H_2O$, $Gd(NO_3)_3 \cdot 6H_2O$; 99.99%) were obtained from Sigma-Aldrich and employed as non-radioactive surrogates. Radioactive tracer solutions of ^{241}Am, ^{244}Cm, and ^{252}Cf were provided by the Institute of Nuclear and New Energy Technology (INET). *Due to the high radioactivity and radiotoxicity, all work involving these radionuclides was conducted in designated radiochemical laboratories under institutional safety protocols, with appropriate shielding, contamination control, personal dosimetry, and radioactive-waste management*. The hydrophilic ligand **Phen-2DIHis** was prepared according to our recent reported methods.[1] The synthesis, purification, and full characterization are therefore not repeated here.

Characterization Methods

The total α-activity concentration of each solution sample was quantified using a liquid scintillation spectrometer (Quantulus 1220, PerkinElmer). Because the liquid scintillation measurement did not provide isotope-specific resolution for the mixed ^{241}Am, ^{244}Cm, and ^{252}Cf tracer samples, the same samples were subsequently analyzed by α-spectrometry (Alpha Analyst 7200-12, Canberra) to determine the fractional contribution of each radionuclide. In the present analytical procedure, LSC provided the quantitative total-activity concentration, whereas α-spectrometry provided isotope discrimination; both measurements were required to obtain the individual radionuclide activity concentrations. The activity concentration of each isotope was then calculated by multiplying the total activity concentration by the corresponding fractional count rate derived from the α-spectrum. UV-vis absorption spectra were recorded at 298 K using a Cary 6000i spectrophotometer (Agilent Inc.) equipped with 1 cm path-length cuvettes. Nuclear Magnetic Resonance spectra (NMR) were acquired on a Varian-600 spectrometer.

Solvent Extractions

A standard liquid-liquid extraction experiment procedure was performed as follows. The aqueous phase was prepared by dissolving a precisely weighed portion of the target precursor hydrophilic ligand (**Phen-2DIHis-Ester** in the present system) in nitric acid solutions and then undergoing complete hydrolysis (the target ligand was **Phen-2DIHis**). As mentioned in Ref 1, to ensure fully hydrolysis of **Phen-2DIHis-Ester**, 50 mM **Phen-2DIHis-Ester** in 1.0 M HNO_3 was preconditioned by heating the solution at 80 °C for 2 h before diluting the solution to desired concentration and acidity (for higher acidity than 1.0 M HNO_3,20 mM solutions of **Phen-2DIHis-Ester** in corresponding acid were heated under the same condition directly). Each aqueous solution was then spiked with trace amounts of the radiotracers ^{241}Am, ^{244}Cm, and ^{252}Cf. The organic phase consisted of **TODGA** dissolved in a mixture of *n*-dodecane (90 vol%) and *n*-octanol (10 vol%). Equal volumes (0.5 mL each) of the aqueous and organic phases were combined in sealed glass vials and equilibrated at 25 ± 1 °C for 120 min under vigorous shaking using a vortex mixer placed in a thermostated water bath. After phase equilibration, the mixtures were centrifuged (3000 rpm, 2 min) to achieve clear phase separation. Samples from both phases were collected before and after extraction. The total activities of ^{241}Am, ^{244}Cm, and ^{252}Cf were quantified with a liquid scintillation spectrometer (Quantulus 1220,

PerkinElmer). The same samples were subsequently analyzed by alpha spectrometry (Alpha Analyst 7200-12, Canberra) to determine the individual count rates of ^{241}Am, ^{244}Cm, and ^{252}Cf. The activity concentration of each isotope was calculated by multiplying the total activity concentration of the sample by the corresponding fractional count rate of specific isotope. The distribution ratio (D_M) for each radionuclide was defined as the ratio of its radioactivity per unit volume in the organic phase to that in the aqueous phase. Separation factors ($SF_{An/An}$) were derived from the ratios of the respective D_M values.

The above process can be expressed mathematically as follows: For each equilibrated organic- and aqueous-phase sample, the total α-activity concentration $A_{\Sigma,p}^{LSC}$ was first measured by liquid scintillation counting, where p denotes the organic or aqueous phase. The same sample was then analyzed by α-spectrometry to obtain the background-corrected count rate, $R_{i,p}^{\alpha}$, of each radionuclide i (^{241}Am, ^{244}Cm, or ^{252}Cf). The isotope-specific fractional count rate was calculated as:

$$f_{i,p}^{\alpha} = \frac{R_{i,p}^{\alpha}}{\sum_j R_{j,p}^{\alpha}}$$

The activity concentration of isotope i in phase p was then obtained from:

$$A_{i,p} = A_{\Sigma,p}^{LSC} f_{i,p}^{\alpha}$$

Accordingly, the isotope-specific distribution ratio was calculated as:

$$D_i = \frac{A_{i,org}}{A_{i,aq}}$$

and the separation factor between radionuclides i and k was calculated as:

$$SF_{i/k} = \frac{D_i}{D_k}$$

In this analytical procedure, liquid scintillation counting and α-spectrometry are both required. Liquid scintillation counting alone measures only the total α-activity of the mixed-radionuclide sample and cannot resolve the individual contributions of ^{241}Am, ^{244}Cm, and ^{252}Cf. Conversely, α-spectrometry was used here to determine the relative isotope composition rather than as an independently calibrated absolute-activity measurement. Direct division of the α-spectrometric count rates obtained from separately prepared organic- and aqueous-phase samples would be valid only if the aliquot volumes, source-preparation recoveries, counting geometries, self-absorption effects, and detector efficiencies were identical or separately calibrated. Therefore, the unnormalized α-spectrometric count-rate ratio was not used to calculate D_i.

Unless otherwise stated, all solvent-extraction experiments were independently performed in triplicate (n = 3). For each replicate, the distribution ratio (D_M) was calculated from the paired activity concentrations measured in the organic and aqueous phases. The separation factor ($SF_{An/An}$) for each

radionuclide pair was then calculated from the corresponding values obtained in the same extraction experiment. The reported values are presented as mean ± standard deviation (SD), and the error bars in all extraction figures represent the SD of the three independent experiments.

UV-vis Absorption Spectroscopy Titrations

The coordination behavior of **Phen-2DIHis** with Nd(III), Sm(III) and Gd(III) ions in nitric acid medium was probed by UV-vis absorption titration. All experiments were performed at 25 ± 1 °C using a temperature-controlled cell holder to minimize thermal artifacts. Spectra were acquired on a Cary 6000i UV-vis-NIR spectrophotometer (Agilent Inc.) with a 1.0 cm quartz cuvette. To maintain a constant ionic background, 0.1 M $NaNO_3$ was added as a supporting electrolyte in all solutions. Titrations were carried out by incremental addition of a 16 mM $Ln(NO_3)_3$ (Ln = Nd, Sm, Gd) solution in 0.01 M HNO_3 to 1.6 mL of a 0.01 mM ligand solution in the same acid medium. After each addition, the mixture was agitated for 5 min at 25 ± 1 °C to ensure equilibration, which confirmed independently by extraction-kinetics studies, before recording the spectrum over 250-370 nm, a region covering the characteristic ligand-based and metal-ligand charge transfer absorption features. Finally, the full spectral dataset was analyzed with *HypSpec* (the non-linear regression software) to extract the derivation of complex-species distributions and the corresponding thermodynamic stability (log β) of each complex species.

Nuclear Magnetic Resonance Spectroscopy (NMR) Titrations

NMR titration experiments were conducted using a Varian-600 spectrometer. All experiments were performed in D_2O containing 1.0 M DNO_3 as the solvent. During titrations, a stock solution of the ligand **Phen-2DIHis-Ester** (25 mM) was prepared as described in the previous section in 1.5 mL of the same solvent to prepare **Phen-2DIHis** and divided equally into three aliquots for independent titrations with Nd(III), Sm(III) and Gd(III) ions. Each titration proceeded through incremental additions of 5–10 µL from corresponding concentrated metal stock solutions (100 mM of $Nd(NO_3)_3$, $Sm(NO_3)_3$ or $Gd(NO_3)_3$ in 1.0 M DNO_3/D_2O). Following every addition, the sample was mixed by thorough inversion of the NMR tube for 5 minutes to achieve equilibrium before spectrum collection. The stability of the free ligand and the formed metal complexes throughout the titration process was verified by consistent tracking of diagnostic NMR signal changes under the acidic conditions employed.

DFT Calculations

All molecular geometries were fully optimized using density functional theory (DFT) with the Gaussian 16, Revision C.01 package at the B3LYP level of theory.[2-4] Harmonic vibrational frequency calculations were performed at the same level to confirm that each optimized structure corresponded to a true minimum on the potential energy surface (i.e., no imaginary frequencies) and to obtain thermal corrections. For the first- and second-row elements C, H, O, and N, the 6-31+G** basis set, including both polarization and diffuse functions, was employed.[5, 6] For americium (Am), curium (Cm), and californium (Cf), a small-core effective core potential (ECP) was used, as it generally provides more reliable results than large-core alternatives, together with the ECP60MWB SEG valence basis set to describe the valence electrons.[7] Because sufficiently reliable solvent-model parameters and dispersion-correction data for Am, Cm, and Cf were unavailable within the Gaussian-based computational framework employed in this work, all calculations were carried out in the gas phase. The same ligand model, coordination stoichiometry, and computational protocol were applied to all three actinide systems to permit an internally consistent comparison of their relative energetic

trends. The calculated ΔG values are not intended to represent quantitative extraction free energies in nitric acid solution because bulk solvation, protonation equilibria, ionic-strength effects, and explicit HNO_3/H_2O interactions were not included. These solution-medium effects may influence both the absolute energies and the magnitudes of the relative energetic differences among the three actinide systems. Gibbs free energies were obtained by combining electronic energies with thermal corrections derived from frequency calculations. To better approximate the standard state in solution, the gas-phase frequency results were converted to the 1 M standard state commonly used in experiments and corrected according to Eq. (1), where Δn denotes the change in the number of species (moles) between products and reactants.[8] The binding energies at bond critical points were estimated using a reliable empirical equation, Eq. (2), for ionic hydrogen bonds, where ΔE is the hydrogen bond energy, and $\rho(r_{BCP})$ is the electron density at the bond critical point (BCP) of the hydrogen bond.[9] Electrostatic potential (ESP) maps,[10] electron localization function (ELF),[11] independent gradient model based on Hirshfeld partition (IGMH),[12] quantum theory of atoms in molecules (QTAIM) topology analysis,[13] and BCP searches were performed using Multiwfn 3.8.1, and the resulting plots were visualized with VMD.[9, 10, 14]

$$\Delta G_{1M}^{0} = \Delta G_{1atm}^{0} + RT \cdot \ln(24.46) \approx \Delta G_{1atm}^{0} + \Delta n \cdot 1.89\ \mathrm{kcal \cdot mol^{-1}}\ (298.15\ \mathrm{K}) \quad \text{Eq. (1)}$$

$$\Delta E \approx -332.34 \times \rho(r_{BCP}) - 1.0661 \quad \text{Eq. (2)}$$

Table S1 Comparison of representative literature reported Am(III)/Cf(III) and Cm(III)/Cf(III) separation performances with the current report.

No.	$SF_{Am(III)/Cf(III)}$	$SF_{Cm(III)/Cf(III)}$	Experimental conditions	References
1	8.0	8.0	[a]O: 1 mM **H_2BP8** in chloroform; [b]A: pH of 5 $HClO_4$ with 1 M $NaClO_4$, O/A = 1	Ref[15]
2	1.2	1.5	O: 0.1 M **TEtDAPhen** in nitrobenzene; A: 3.0 M HNO_3; O/A = 1	Ref[16]
3	4	7.2	O: 0.005 M **C5-BTBP** in cyclohexanone; A: 0.5 M HNO_3 with 0.5 M $NaNO_3$; O/A = 1	Ref[17]
4	NA[c]	3.34	O: 0.1 M **NTAamide(C8)** in kerosene; A: 0.1 M HNO_3; O/A = 1	Ref[18]
5	6.5	1.5	O: 0.4 M **$(ClPh)_2$PSSH** + 0.15 M **TEHP** in 20% isooctane/80% *tert* - butyl benzene A: 0.13 M HNO_3; O/A = 1	Ref[19]
6	6.7	6.0	O: 0.2 M **BMPPT** and 0.04 M **TOPO** in toluene; A: 0.1 M $NaClO_4$ and 0.01 M sulfanilic acid, pH = 3.5; O/A = 1	Ref[20]
7	NA[c]	3.2	O: 0.12 M **HDEHP** in toluene; A: 0.01 M **2-methyllactic acid** in 0.1 M HCl; O/A = 1	Ref[21]
8	NA[c]	2.9	O: 0.12 M **HDEHP** in toluene; A: 0.01 M **citric acid** in 0.1 M HCl; O/A = 1	
9	NA[c]	4.4	O: 0.12 M **HDEHP** in toluene; A: 0.01 M **malic acid** in 0.1 M HCl; O/A = 1	
10	NA[c]	4.5	O: 0.12 M **HDEHP** in toluene; A: 0.01 M **tartaric acid** in 0.1 M HCl; O/A = 1	
11	6.8	6.1	O: 0.3 M **HDEHP** in diisopropylbenzene; A: 1.0 M lactic acid with 0.05 M **DTPA** (pH of 3); O/A = 1	Ref[22]
12	5.1	4.5	O: 0.5 M **HDEHP** in decane; A: 1.0 M **lactic acid** with 0.07 M Na_5DTPA; O/A = 1	Ref[23]
**13**	**383**	**36.9**	O: 0.1 M **TODGA** in *n*-dodecane/*n*-octanol (90/10, v/v); A: 1.0 M HNO_3 with 25 mM **Phen-2DIHis**; O/A = 1	**This work**

[a] Organic phase. [b] Aqueous phase. [c] Not available.

The blue shaded region indicates both ligands were used in the organic and aqueous phases and the grey region is the data from current report.

Table S2 Comparison of representative literature reported Am(III)/Cm(III) separation performances by liquid-based extraction.

No.	$SF_{Am(III)/Cm(III)}$	Experimental conditions	References
1	<1.4	[a]O: 30% **TBP** in kerosene;<br>[b]A: 0-6 M HNO_3, O/A = 1	Ref[24]
2	1.2-1.5	O: 5% **TOPO** in xylene;<br>A: 0.01-0.5 M HNO_3/NH_4NO_3; O/A = 1	Ref[25]
3	<1.45	[c]S: **CMPO** as extraction chromatography sorbent;<br>A: 0.01-10 M HNO_3;	Ref[26]
4	1.1-1.5	O: 0.65 M **DMDOHEMA** in TPH (hydrogenated tetrapropylene);<br>A: 0.5-2.5 M HNO_3; O/A = 1	Ref[27]
5	1.2	O: 0.2M **TODGA** in TPH with 5% 1-octanol;<br>A: 1.0 M HNO_3; O/A = 1	Ref[28]
6	5.5	O: 0.2 M **ADAAM** in *n*-dodecane;<br>A: 1.5 M HNO_3; O/A = 1	Ref[29]
7	2.2-2.8	O: 0.1 mol/L **Et(pHexPh)Dpy** in F-3;<br>A: 0.5-5.0 M HNO_3; O/A = 1<br>（0.5/2.0M-2.8；5.0M-2.2）	Ref[30]
8	1.1-1.3	O: 0.001 mol/L ***t*BuPhPyPO** in nitrobenzene;<br>A: 0.1-3.0 M HNO_3; O/A = 1	Ref[31]
9	2.9-3.5	O: 0.001 mol/L **Ph_2PyPO** in nitrobenzene;<br>A: 0.1-3.0 M HNO_3; O/A = 1	
10	~1	O: 0.03 M **$CyMe_4$-BTPhen** in 1-octanol;<br>A: 0.1-3.0 M HNO_3; O/A = 1	Ref[32]
11	2-7	O: 0.03 M **5-Br-$CyMe_4$-BTPhen** in 1-octanol;<br>A: 0.1-3.0 M HNO_3; O/A = 1	
12	2-5	O: 0.03 M **5-(4-hydroxyphenyl)-$CyMe_4$-BTPhen 5** in 1-octanol;<br>A: 0.1-3.0 M HNO_3; O/A = 1	
13	1.67-1.85	O: 0.1-0.5 M **NTAamaide** in *n*-dodecane;<br>A: 0.2 M HNO_3; O/A = 1	Ref[33]
14	2.45	O: 0.1 M **DOODA** in *n*-dodecane;<br>A: 3.0 M HNO_3; O/A = 1	Ref[34]
15	1.4	O: 0.6 M **DMDOHEMA** + 0.45 M HDEHP in TPH;<br>A: 6.0 M HNO_3; O/A = 1	Ref[35]
16	1.2	O: 20 mM of **TODGA** in 1-octanol/TPH 40/60 $\%_{vol.}$;<br>A: 1.0 M HNO_3; O/A = 1	
17	1.5	O: 20 mM of **TnBDGA** in 1-octanol/TPH 40/60 $\%_{vol.}$;<br>A: 1.0 M HNO_3; O/A = 1	
18	1.3	O: 20 mM of **TiBDGA** in 1-octanol/TPH 40/60 $\%_{vol.}$;<br>A: 1.0 M HNO_3; O/A = 1	
19	1.1	O: 20 mM of **TsBDGA** in 1-octanol/TPH 40/60 $\%_{vol.}$;<br>A: 1.0 M HNO_3; O/A = 1	
20	2.6	O: 0.01 M **TEDGA** in C4mim.Tf2N;	Ref[36]

		A: 3.0 M HNO_3; O/A = 1	
21	1-1.8	O: 0.01 mol/L **$CyMe_4BTPhen$** in 1-octanol;<br>A: 0.001-3.0 M HNO_3; O/A = 1	Ref[37]
22	1.66-6.5	O: 0.1-0.5 M **NTAamaide** in *n*-dodecane;<br>A: 10 mM **TEDGA** in 0.2 M HNO_3; O/A = 1	Ref[33]
23	1.8	O: 0.6 M **DMDOHEMA** + 0.45 M HDEHP in TPH;<br>A: 30 mM **TMDGA** in 6.0 M HNO_3; O/A = 1	Ref[35]
24	2.3	O: 0.6 M **DMDOHEMA** + 0.45 M HDEHP in TPH;<br>A: 30 mM **TEDGA** in 6.0 M HNO_3; O/A = 1	
25	1.0	O: 0.6 M **DMDOHEMA** + 0.45 M HDEHP in TPH;<br>A: 30 mM **TnDGA** in 6.0 M HNO_3; O/A = 1	
26	3.9	O: 0.01 mol/L **$CyMe_4BTPhen$** in Aliquat-336 Nitrate Ionic Liquid;<br>A: 0.01 mol/L **TEDGA** in 0.05 M HNO_3; O/A = 1	Ref[38]
27	~1	O: 0.2 mol/L **TODGA** + 5 % vol. 1-octanol in TPH;<br>A: 9 mM **SO_3-Ph-BTP** in 0.1-1.0 M HNO_3; O/A = 1	Ref[39]
28	2.4–3.6	O: 0.2 mol/L **TODGA** + 5 % vol. 1-octanol in Exxsol D80;<br>A: 20 mM **SO_3-Ph-BTBP** in 0.2-1.5 M HNO_3; O/A = 1	Ref[40]
29	2.5–4.5	O: 0.2 mol/L **TODGA** + 5 % vol. 1-octanol in TPH;<br>A: 10 mM **TS-BTPhen** in 0.01-1.0 M HNO_3; O/A = 1	Ref[41]
30	6.5	O: 0.05 M **Hdehp** in o-xylene;<br>A: 0.001 M **H_2bp18c6** in 0.001 M HNO_3 with 1 M $NaNO_3$	Ref[42]
31	2.53-3.13	O: 0.1 M **TODGA** in TPH;<br>A: 0-1.3 mM **H4TPAEN** in 0.1 M HNO_3 with 1 M $NaNO_3$	Ref[43]
**32**	**10.4**	O: 0.1 M **TODGA** in *n*-dodecane/*n*-octanol (90/10, v/v);<br>A: 1.0 M HNO_3 with 25 mM **Phen-2DIHis**; O/A = 1	**This work**

[a] Organic phase. [b] Aqueous phase.

The blue shaded region indicates both ligands were used in the organic and aqueous phases and the grey region is the data from current report.

Supporting Information Note 1

In the current reported biphasic system, after extraction reached extraction equilibrium (pre-experiment was conducted by measuring the metal distribution as a function of time), the distribution ratio (D_M) of a specific metal ion (M) between the aqueous and organic phases was defined as the ratio of the concentration in the organic phase (C_{org}) to that in the aqueous phase (C_{aq}):

$$D_M = C_{org}/C_{aq}$$

The separation factor ($SF_{An/An}$) was used to evaluate the efficacy of the extraction system in separating the two metal ions and was defined as the ratio of their distribution ratios:

$$SF_{An/An} = D_{M1}/D_{M2}$$

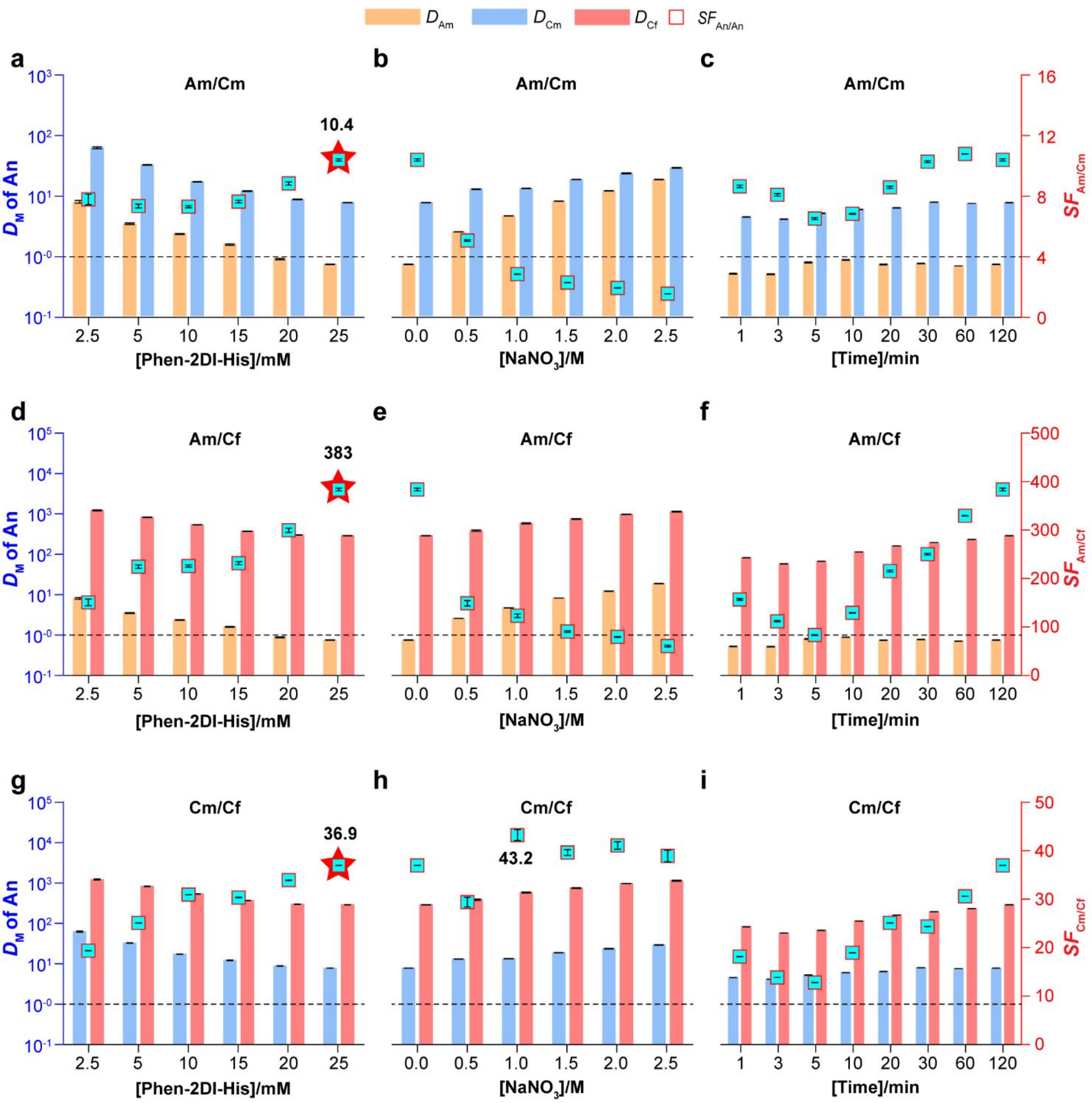

Figure S1. **Experimental conditions screening for Am(III), Cm(III) and Cf(III) separation with Phen-2DIHis/TODGA system.** Extraction performance for Am(III)/Cm(III) (a-c), Am(III)/Cf(III) (d-f) and Cm(III)/Cf(III) (g-i) as functions of ligand concentrations, $NaNO_3$ concentrations and contacting times. Color codes were distribution ratios (D_M) of Am(III) (orange), Cm(III) (blue) and Cf(III) (red), and cyan open squares denote the corresponding separation factors ($SF_{An/An}$). Experimental conditions (a, d, g): 0.1 M **TODGA** in *n*-dodecane/*n*-octanol (90/10, v/v) as the organic phase, O; the aqueous phase consisted of 1.0 M HNO_3 with **Phen-2DIHis** concentrations in the range of 2.5 mM to 25 mM; T=298 K and O/A = 1:1 for 120 minutes. Data are presented as mean ± standard deviation from three independent extraction experiments (n = 3); error bars represent standard deviation.

Experimental conditions (b, e, h): 0.1 M **TODGA** in *n*-dodecane/*n*-octanol (90/10, v/v) as the organic phase, O; the aqueous phase consisted of 1.0 M HNO_3 with 25 mM **Phen-2DIHis** and $NaNO_3$ of different concentrations; T=298 K and O/A = 1:1 for 120 minutes.

Experimental conditions (c, f, i): 0.1 M **TODGA** in *n*-dodecane/*n*-octanol (90/10, v/v) as the organic phase, O; the aqueous phase consisted of 1.0 M HNO_3 with 25 mM **Phen-2DIHis**; T=298 K and O/A = 1:1 for different contacting times.

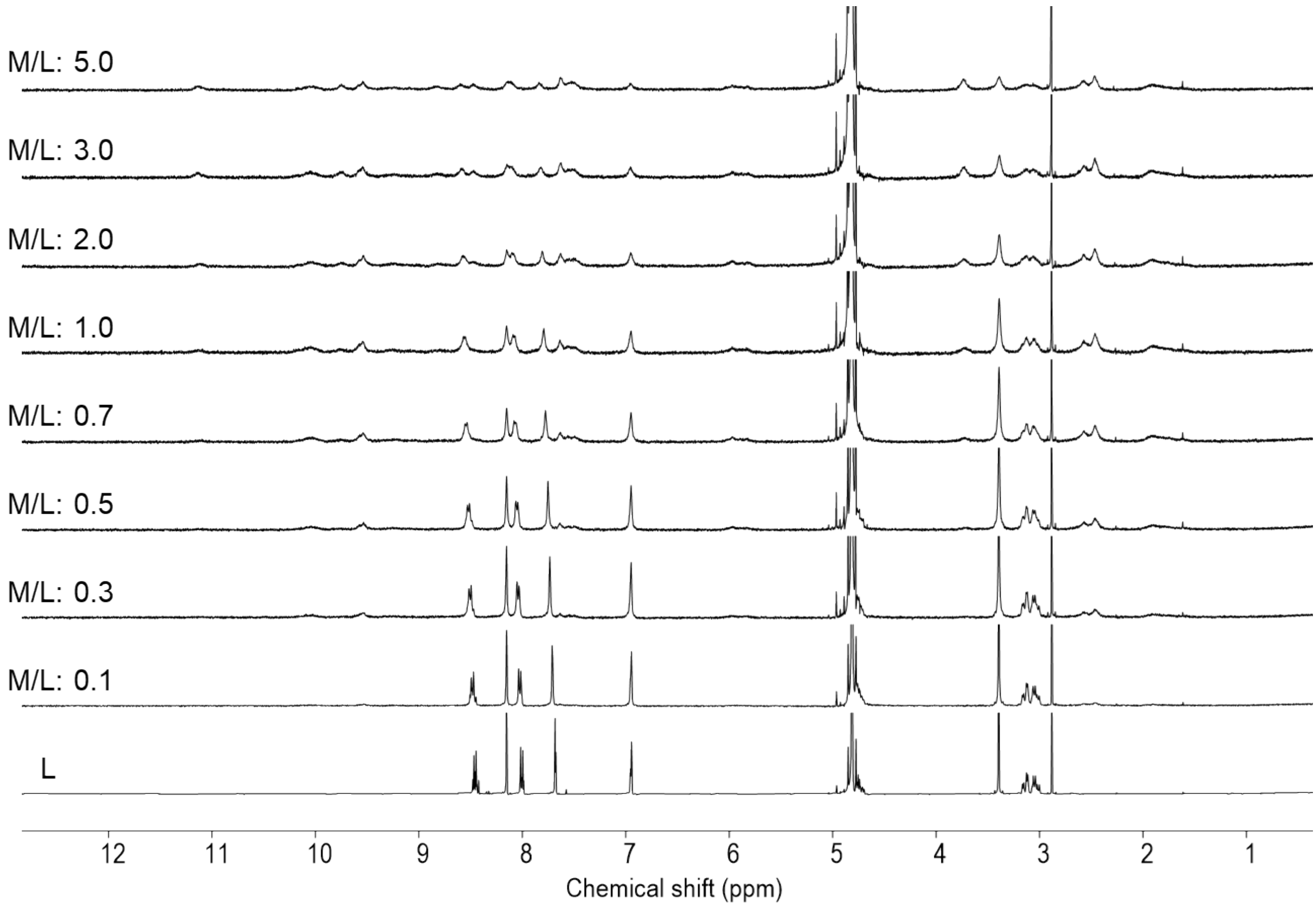

Figure S2. Stacked ^1H NMR spectra of **Phen-2DIHis** titrated with $Nd(NO_3)_3$.

Experiment conditions: 25 mM **Phen-2DIHis** in 1.0 M DNO_3/D_2O titrated with 0.1 M $Nd(NO_3)_3$ at 25 °C. Metal/ligand ratios were given on the left side of figure.

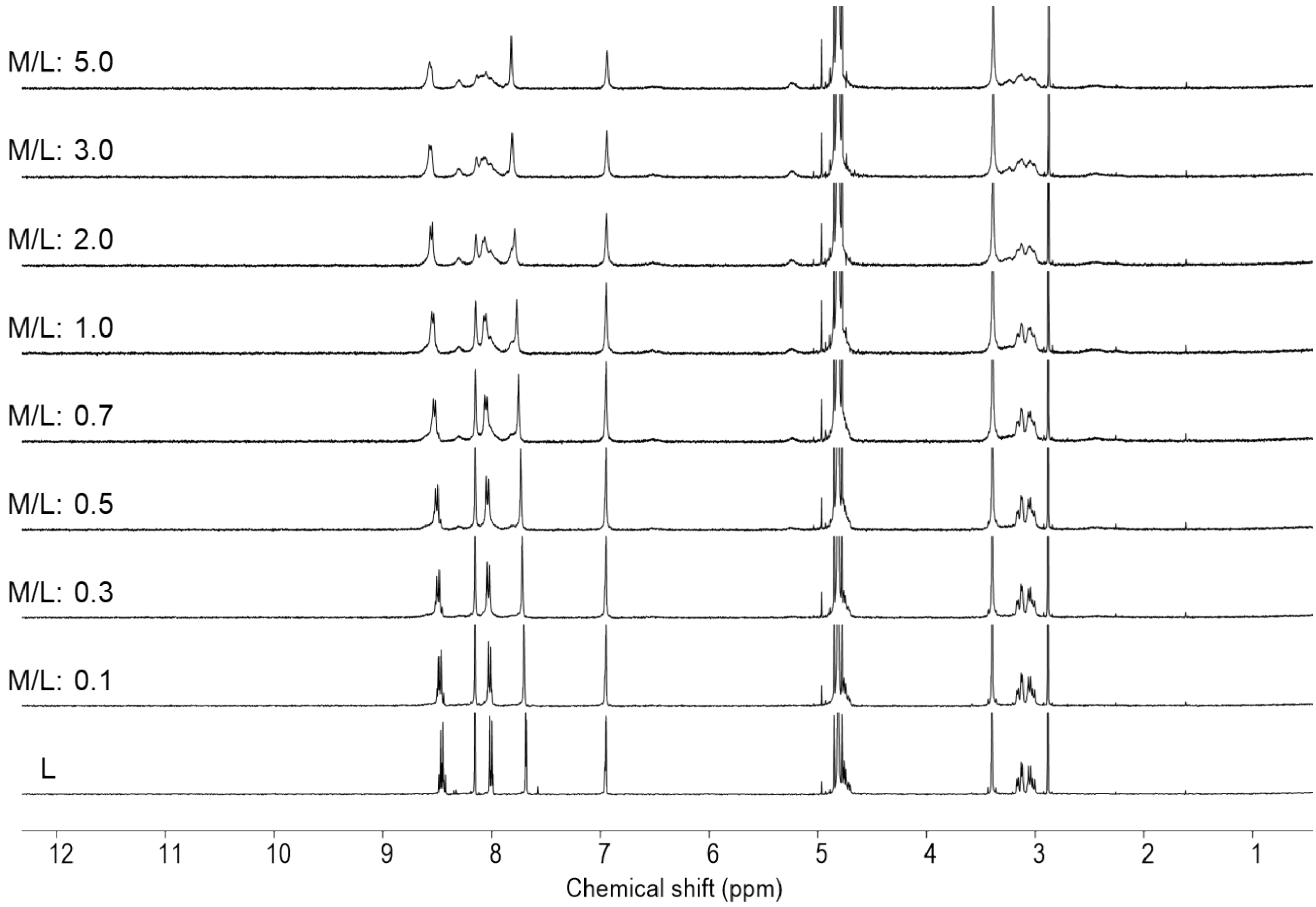

Figure S3. Stacked ^{1}H NMR spectra of **Phen-2DIHis** titrated with $Sm(NO_3)_3$.

Experiment conditions: 25 mM **Phen-2DIHis** in 1.0 M DNO_3/D_2O titrated with 0.1 M $Sm(NO_3)_3$ at 25 °C. Metal/ligand ratios were given on the left side of figure.

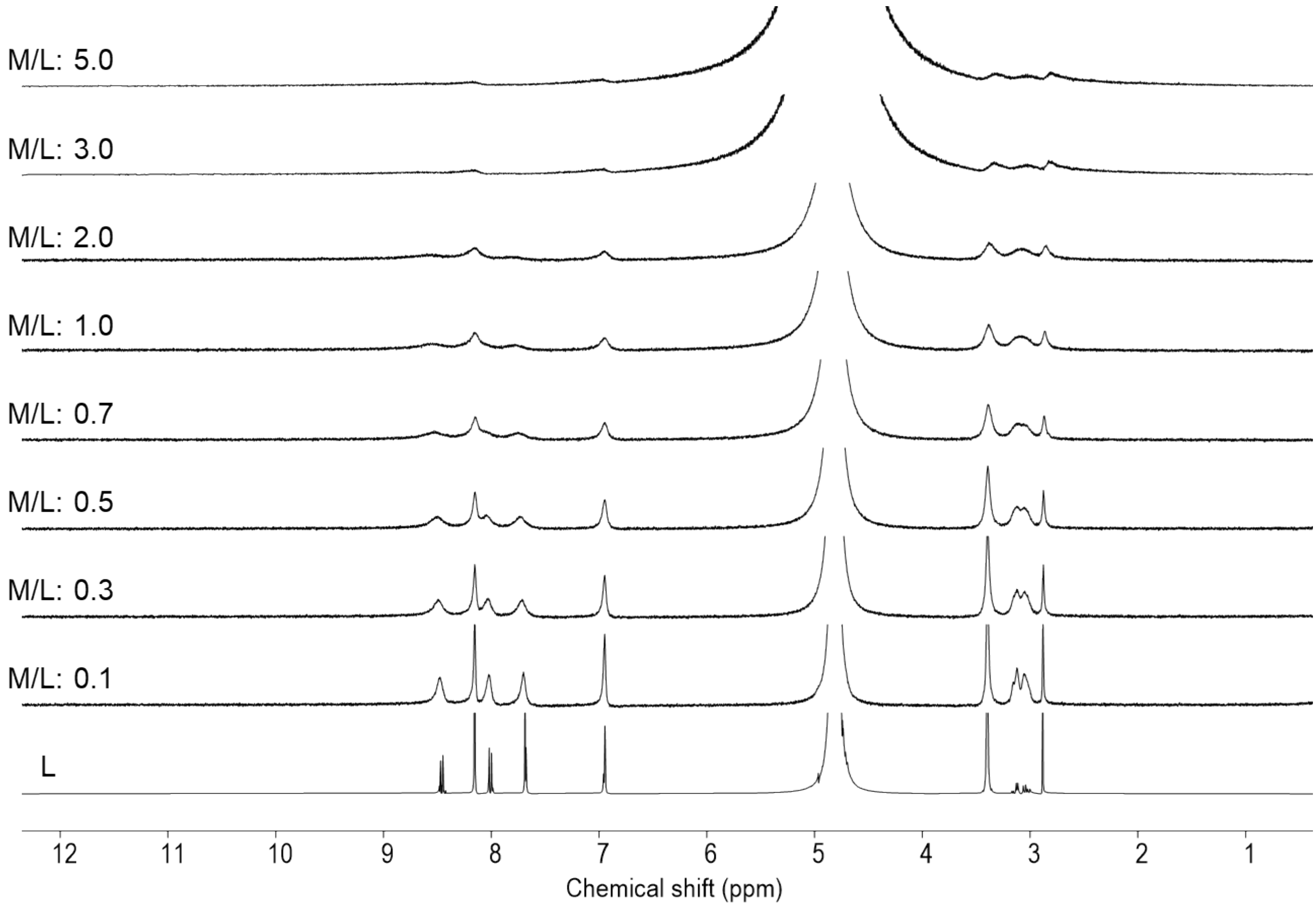

Figure S4. Stacked ^{1}H NMR spectra of **Phen-2DIHis** titrated with $Gd(NO_3)_3$.

Experiment conditions: 25 mM **Phen-2DIHis** in 1.0 M DNO_3/D_2O titrated with 0.1 M $Gd(NO_3)_3$ at 25 ℃. Metal/ligand ratios were given on the left side of figure.

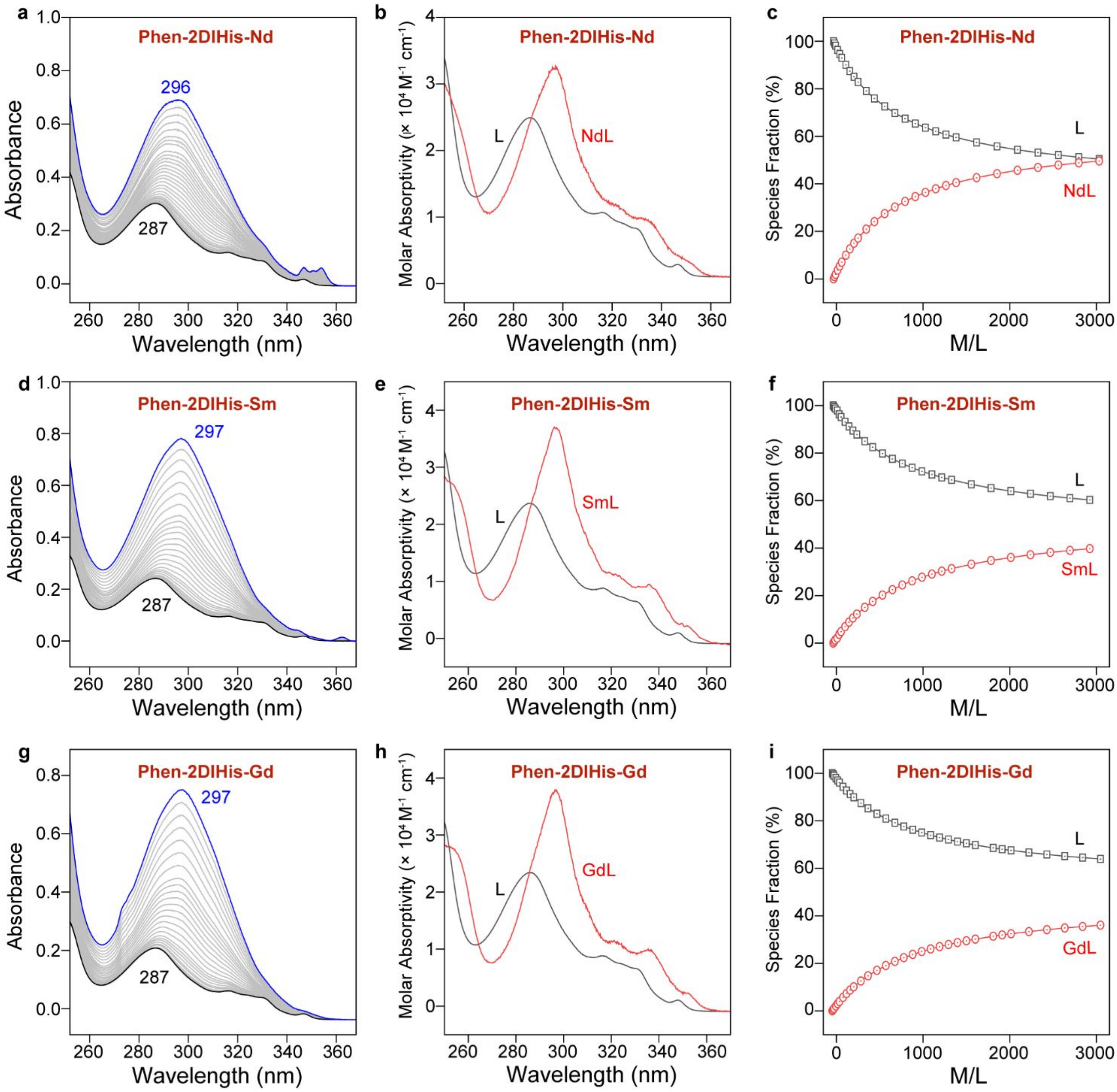

Figure S5. Absorption spectra titrations of **Phen-2DIHis** with Nd(III) (a), Sm(III) (d), and Gd(III) (g). Black and blue traces represented the spectra before and after the titration with main peaks marked on the figure.

Experiment conditions: 0.01 mM **Phen-2DIHis** in 0.01 M HNO_3 titrated with 16 mM $Nd(NO_3)_3$, $Sm(NO_3)_3$, or $Gd(NO_3)_3$, ion strengths controlled by 0.1 M $NaNO_3$. Speculated species molar absorptivity (b, e and h) and species evolutions (c, f and i) during titration were deduced from panel (a, d and g).

Supporting Information Note 2

During the optimization of the complex geometries, three converged configurations were obtained for each An(III)-**Phen-2DIHis** complex (Fig. S6). Further energy calculations showed that configuration b had the lowest electronic energy and was therefore thermodynamically the most stable. Accordingly, configuration b was selected for the subsequent calculations of the Am(III), Cm(III), and Cf(III) complexes with **Phen-2DIHis**. In all **Phen-2DIHis**-An(III) models, the two terminal carboxyl groups were represented in their protonated, neutral -COOH form, whereas each imidazole ring was modeled in its neutral form, containing one pyridine-type nitrogen and one pyrrole-type N-H nitrogen. This neutral-imidazole structure was adopted as a common reference model for Am(III), Cm(III), and Cf(III), rather than as the predominant microspecies in 1.0 M HNO_3. Explicit protonation of both imidazole groups would introduce two additional positive charges and require additional nitrate counterions. Because the locations, ion-pairing modes, and hydration configurations of these counterions cannot be uniquely defined within the present static gas-phase model, incorporating a single protonated-counterion structure would introduce configuration-dependent electrostatic and hydrogen-bonding contributions. The neutral-imidazole model was therefore retained to permit an internally consistent comparison of relative metal-dependent trends under identical conditions. The calculated structures and energies should not be interpreted as a protonation-state-resolved description of the dominant solution species.

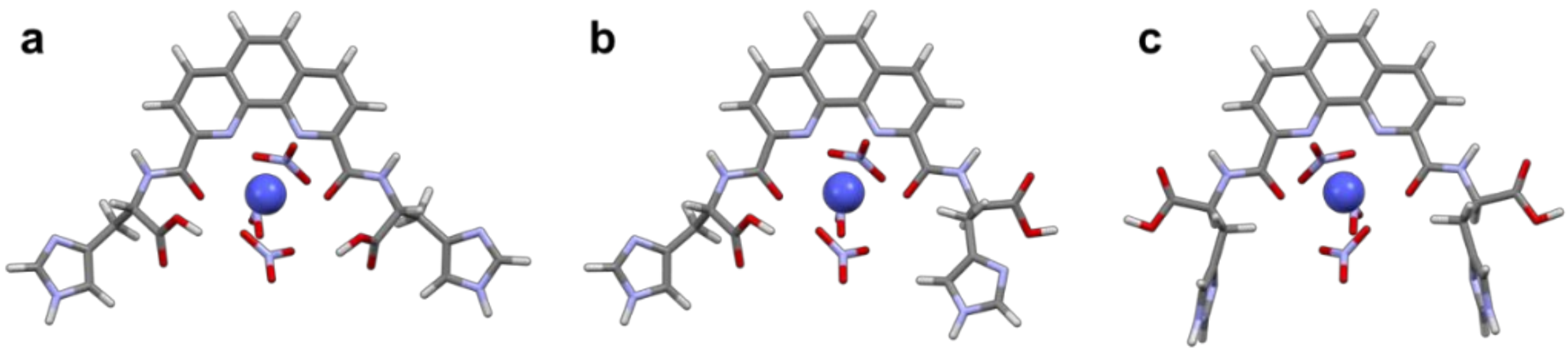

Figure S6. The three configurations of the **Phen-2DIHis**-Am(III) complex after geometric optimization. The terminal carboxyl groups were modeled as neutral -COOH groups, whereas the imidazole rings were modeled as neutral N-H groups. This structure represents the common neutral reference model used for relative Am(III)/Cm(III)/Cf(III) comparison.

Supporting Information Note 3

TEDGA was employed as a truncated computational model of **TODGA**. Both ligands possess the same tridentate diglycolamide *O*, *O*, *O* donor core, whereas the peripheral *N*-alkyl substituents are ethyl groups in **TEDGA** and *n*-octyl groups in **TODGA**. Truncation of the *N*-alkyl chains substantially reduces molecular size and conformational complexity while preserving the local metal-binding motif. The alkyl-chain substitution may influence absolute solvation, conformational, aggregation, and phase-transfer energetics; therefore, **TEDGA** is not regarded as a complete representation of the organic-phase behavior of **TODGA**. However, the same **TEDGA** model, coordination stoichiometry, and computational protocol were applied consistently to Am(III), Cm(III), and Cf(III), and the resulting energies are used only to compare their relative trends. Similar combinations of **TEDGA**-based DFT models and experimental or molecular-dynamics descriptions of full **TODGA** extraction systems have been employed in previous high-level studies of **DGA** coordination and selectivity.[44, 45]

Table S3. Binding energies of **Phen-2DIHis** with Am(Ⅲ), Cm(Ⅲ), and Cf(Ⅲ) in nitric acid system.

Type	Reaction	ΔG ($kcal \cdot mol^{-1}$)
Binding energy	**Phen-2DIHis** + $[Am(H_2O)_8NO_3]^{2+}$ + $2NO_3^-$ → [**Phen-2DIHis**]$[Am(NO_3)_3]$ + $(H_2O)_8$	-263.16
Binding energy	**Phen-2DIHis** + $[Cm(H_2O)_8NO_3]^{2+}$ + $2NO_3^-$ → [**Phen-2DIHis**]$[Cm(NO_3)_3]$ + $(H_2O)_8$	-261.21
Binding energy	**Phen-2DIHis** + $[Cf(H_2O)_7NO_3]^{2+}$ + $2NO_3^-$ → [**Phen-2DIHis**]$[Cf(NO_3)_3]$ + $(H_2O)_7$	-261.11

Table S4. The Gibbs free energy change (*Δ*G) value of the competitive extraction reaction in this work.

Type	Reaction	ΔG ($kcal \cdot mol^{-1}$)
Binding energy	3**TEDGA** + [**Phen-2DIHis**]$[Am(NO_3)_3]$ → [**TEDGA**]$_3$[Am(III)]$(NO_3)_3$ + **Phen-2DIHis**	-12.79
Binding energy	3**TEDGA** + [**Phen-2DIHis**]$[Cm(NO_3)_3]$ → [**TEDGA**]$_3$[Cm(III)]$(NO_3)_3$ + **Phen-2DIHis**	-13.97
Binding energy	3**TEDGA** + [**Phen-2DIHis**]$[Cf(NO_3)_3]$ → [**TEDGA**]$_3$[Cf(III)]$(NO_3)_3$ + **Phen-2DIHis**	-14.59

Table S5. The calculated bond lengths for Am(III), Cm(III), and Cf(III) with **Phen-2DIHis** at B3LYP/RECP/6-31G(d) level.

Bond Length						
Ligand	Actinide ion	M-O1	M-O2	M-N1	M-N2
**Phen-2DIHis**	Am(III)	2.532	2.515	2.729	2.713
		2.524		2.721	
	Cm(III)	2.527	2.482	2.770	2.729
		2.505		2.749	
	Cf(III)	2.481	2.446	2.732	2.689
		2.464		2.711	

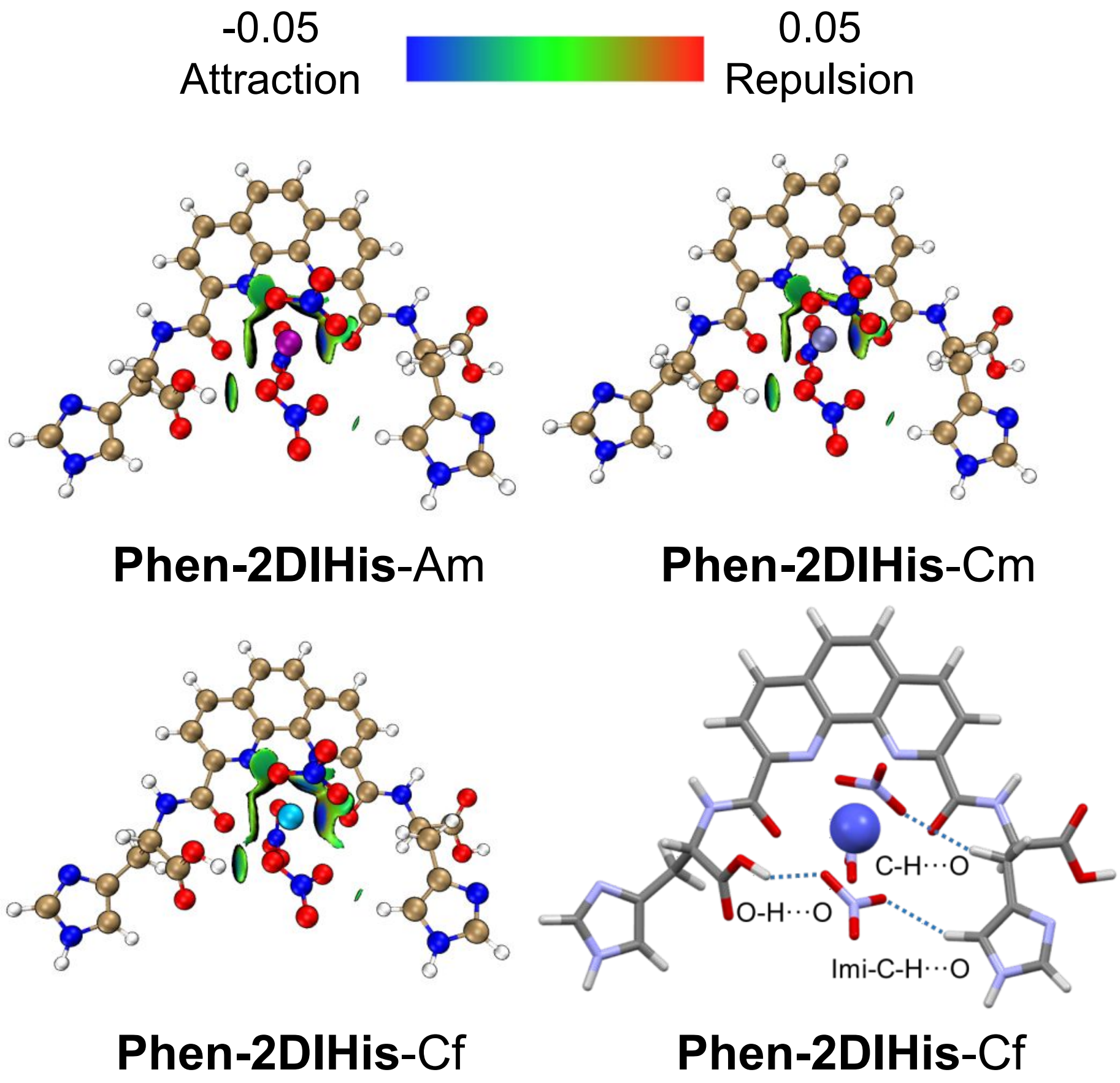

Figure S7. The IGMH (Independent Gradient Model based on Hirshfeld partition) and hydrogen bonding interaction sites in **Phen-2DIHis**-An(III) complexes.

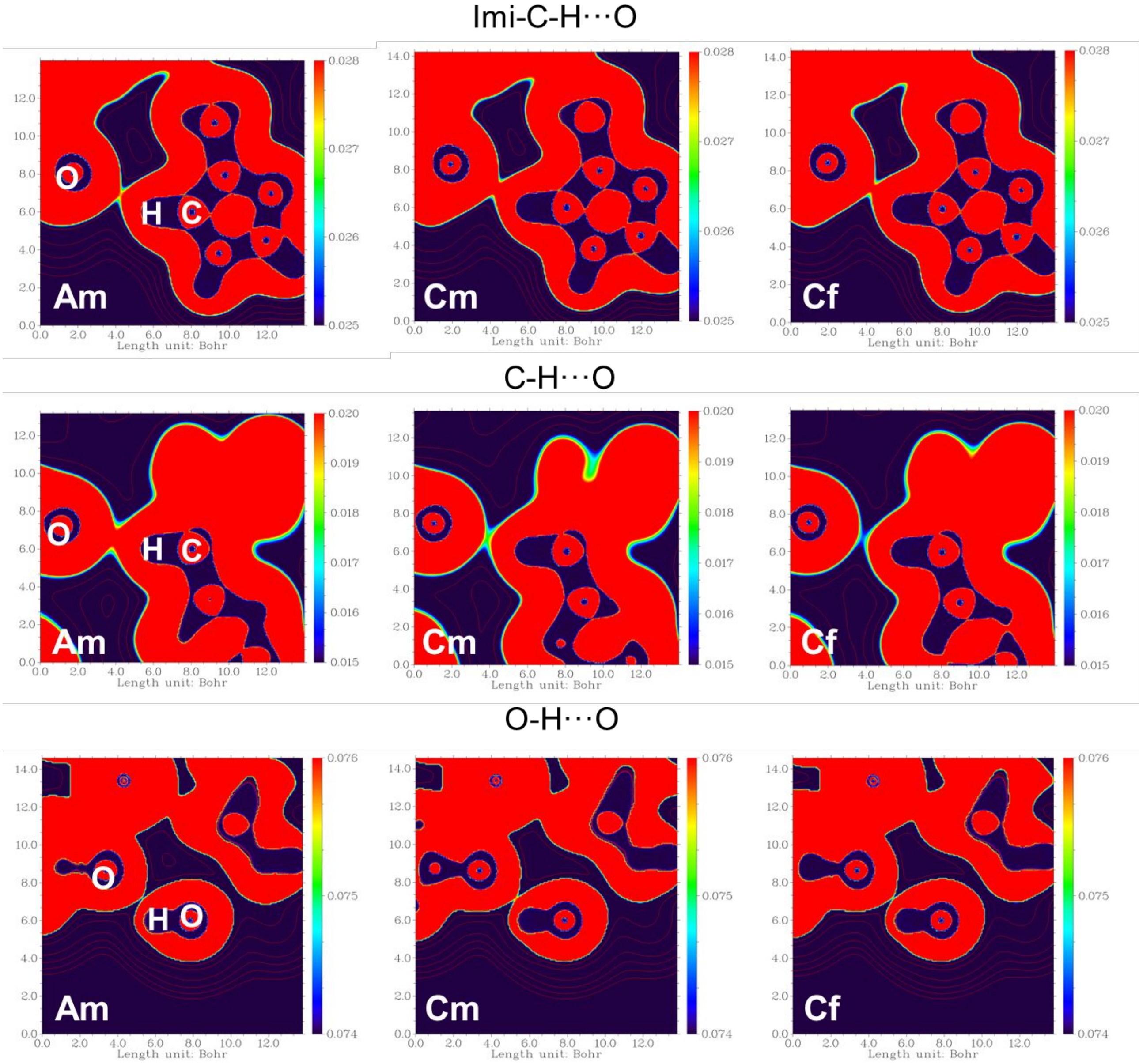

Figure S8. Two-dimensional colour map of the ELF of hydrogen bonds in the **Phen-2DIHis**-An(III) complexes.

Supporting Information Note 4

The one-dimensional ELF profiles along the D-H···A hydrogen-bond direction show a characteristic variation: the ELF value first increases from the donor atom (O) toward the hydrogen atom, then decreases near the H nucleus, and finally reaches a local minimum between the H atom and the acceptor atom (A). This minimum corresponds to the core-valence bifurcation (CVB) value. A lower CVB value indicates a more pronounced electrostatic contribution to the hydrogen bond. In the present systems, all calculated CVB values are relatively low (<0.08), suggesting that these hydrogen bonds are predominantly electrostatic in nature. To further quantify the contribution of hydrogen bonding in the side-arm regions of different metal complexes, the electron densities at the bond critical points (BCPs) of these hydrogen bonds were calculated and used to estimate the ionic hydrogen-bond energies according to Eq. 1. The corresponding BCP electron densities are summarized in Table S6, and the calculated energies of the three representative hydrogen bonds in different metal complexes are shown in Fig. 5. These hydrogen-bond energies reflect the contribution of the hydrogen-bonding network to the Gibbs free energy change (ΔG) of the reaction. Notably, during the competitive extraction of **Phen-2DIHis** and **TODGA**, disruption of these hydrogen-bond interactions would require a corresponding thermodynamic cost.

$$\Delta E \approx -332.34 \times \rho(r_{BCP}) - 1.0661 \qquad \text{eq. 1}$$

Here, ΔE represents the hydrogen bond energy, and ρ(rBCP) is the electron density of the hydrogen bond in BCP.

Table S6. The electron density value of the intramolecular hydrogen bond BCP in the complex molecule.

H-bond	**Phen-2DIHis**-Am(III)	**Phen-2DIHis**-Cm(III)	**Phen-2DIHis**-Cf(III)
Imi-C-H···O	0.00835	0.00894	0.00860
C-H···O	0.00556	0.00451	0.00399
O-H···O	0.02280	0.02315	0.02283

Supporting Information Note 5

HFIR refers to the High Flux Isotope Reactor at Oak Ridge National Laboratory, where curium-containing targets are irradiated for the production of Cf and other transplutonium isotopes. The HFIR-relevant simulated feed used in this study was not intended to reproduce the complete composition of an actual post-irradiation process solution. Instead, it was constructed as a simplified, high-loading trivalent-ion interference matrix to evaluate the influence of residual lanthanides and chemically similar matrix ions on Am(III)/Cm(III)/Cf(III) separation. The solution contained La(III), Ce(III), Pr(III), Nd(III), Sm(III), Eu(III), Gd(III), Tb(III), Dy(III), Ho(III), Er(III), Tm(III), Yb(III), Lu(III), Sc(III), and Y(III), each at a concentration of 10 mM as the corresponding nitrate salt, in 1.0 M HNO_3.

The composition of the simulated liquid named HLLW is as shown in Table S7:

Table S7. Component list of the liquid material HLLW (with burnup of 33,000 MWd/tU, Am component in the TRPO process flow)[46, 47]

Element	Concentration（g/L）	Corresponding nitrate
Eu	0.0151	$Eu(NO_3)_3 \cdot 6H_2O$
La	0.144	$La(NO_3)_3 \cdot 6H_2O$
Mo	0.006575	$(NH_4)_6Mo_7O_{24} \cdot 6H_2O$
Ce	0.281	$Ce(NO_3)_3 \cdot 6H_2O$
Nd	0.476	$Nd(NO_3)_3 \cdot 6H_2O$
Pd	0.163	$Pd(NO_3)_3$
Ru	0.132	$Ru(NO_3)_3$
Rh	0.16125	$Rh(NO_3)_3$
Sm	0.00945	$Sm(NO_3)_3 \cdot 6H_2O$
Y	0.097	$Y(NO_3)_3 \cdot 6H_2O$
Fe	0.007225	$Fe(NO_3)_3 \cdot 6H_2O$
HNO_3	1.075 M	

SI references:

1. Li, B.; Wang, L.; Kang, Y.; Cao, H.; Liu, Y.; He, Q.; Li, Z.; Tang, X.; Chen, J.; Wang, L.; Xu, C., Amino Acid Decorated Phenanthroline Diimide as Sustainable Hydrophilic Am(III) Masking Agent with High Acid Resistance. *JACS Au.* **2024,** *4*, 3668-3678.

2. Kohn, W.; Sham, L. J., Self-Consistent Equations Including Exchange and Correlation Effects. *Phys. Rev* **1965,** *140*, A1133-A1138.

3. Lee, C.; Yang, W.; Parr, R. G., Development of the Colle-Salvetti correlation-energy formula into a functional of the electron density. *Phys. Rev. B* **1988,** *37*, 785-789.

4. Becke, A. D., Density-functional thermochemistry. III. The role of exact exchange. *J. Chem. Phys.* **1993,** *98*, 5648-5652.

5. Hehre, W. J.; Ditchfield, R.; Pople, J. A., Self-Consistent Molecular Orbital Methods. XII. Further Extensions of Gaussian-Type Basis Sets for Use in Molecular Orbital Studies of Organic Molecules. *J. Chem. Phys.* **1972,** *56*, 2257-2261.

6. Hariharan, P. C.; Pople, J. A., The influence of polarization functions on molecular orbital hydrogenation energies. *Theoretica chimica acta* **1973,** *28*, 213-222.

7. Cao, X.; Dolg, M.; Stoll, H., Valence basis sets for relativistic energy-consistent small-core actinide pseudopotentials. *J. Chem. Phys.* **2003,** *118*, 487-496.

8. Skyner, R. E.; McDonagh, J. L.; Groom, C. R.; van Mourik, T.; Mitchell, J. B. O., A review of methods for the calculation of solution free energies and the modelling of systems in solution. *Phys. Chem. Chem. Phys.* **2015,** *17*, 6174-6191.

9. Emamian, S.; Lu, T.; Kruse, H.; Emamian, H., Exploring Nature and Predicting Strength of Hydrogen Bonds: A Correlation Analysis Between Atoms-in-Molecules Descriptors, Binding Energies, and Energy Components of Symmetry-Adapted Perturbation Theory. *J. Comput. Chem.* **2019,** *40*, 2868-2881.

10. Lu, T.; Chen, F., Multiwfn: A multifunctional wavefunction analyzer. *J. Comput. Chem.* **2012,** *33*, 580-592.

11. Becke, A. D.; Edgecombe, K. E., A simple measure of electron localization in atomic and molecular systems. *J. Chem. Phys.* **1990,** *92*, 5397-5403.

12. Lu, T.; Chen, Q., Independent gradient model based on Hirshfeld partition: A new method for visual study of interactions in chemical systems. *J. Comput. Chem.* **2022,** *43*, 539-555.

13. Bader, R. F. W., A quantum theory of molecular structure and its applications. *Chem. Rev.* **2002,** *91*, 893-928.

14. Humphrey, W.; Dalke, A.; Schulten, K., VMD: Visual molecular dynamics. *Journal of Molecular Graphics* **1996,** *14*, 33-38.

15. Takeishi, H.; Kitatsuji, Y.; Kimura, T.; Meguro, Y.; Yoshida, Z.; Kihara, S., Solvent extraction of uranium, neptunium, plutonium, americium, curium and californium ions by bis(1-phenyl-3-methyl-4-acylpyrazol-5-one) derivatives. *Anal. Chim. Acta.* **2001,** *431*, 69-80.

16. Archer, E. M.; Pereiro, F. A.; Riley, J. M.; Brannon, J. P.; Flynn, E. B.; Gilhula, J. C.;

Huffman, B. L.; Jackson, J. A.; Knapp, J. G.; Long, B. N.; Mason, H. E.; Mullis, M. S.; Kozimor, S. A.; Shafer, J. C.; Galley, S. S., The complexation and solvent extraction properties of a phenanthroline diamide extractant for trivalent actinide and lanthanide ions. *Inorg. Chem. Front.* **2025,** *12*, 7421-7432.

17. Nilsson, M.; Ekberg, C.; Foreman, M.; Hudson, M.; Liljenzin, J. O.; Modolo, G.; Skarnemark, G., Separation of Actinides(III) from Lanthanides(III) in Simulated Nuclear Waste Streams using 6,6′-Bis-(5,6-dipentyl-[1,2,4]triazin-3-yl)-[2,2′]bipyridinyl (C5-BTBP) in Cyclohexanone. *Solvent Extr. Ion Exch.* **2006,** *24*, 823-843.

18. Wang, Y.-l.; Li, F.-f.; Xiao, Z.; Wang, C.-z.; Liu, Y.; Shi, W.-q.; He, H., Experimental and theoretical studies on the extraction behavior of Cf(iii) by NTAamide(C8) ligand and the separation of Cf(iii)/Cm(iii). *RSC Adv.* **2023,** *13*, 3781-3791.

19. Modolo, G.; Kluxen, P.; Geist, A., Demonstration of the LUCA process for the separation of americium(III) from curium(III), californium(III), and lanthanides(III) in acidic solution using a synergistic mixture of bis(chlorophenyl)dithiophosphinic acid and tris(2-ethylhexyl)phosphate. *Radiochim. Acta* **2010,** *98*, 193-201.

20. Hannink, N. J.; Hoffman, D. C.; Smith, B. F., EXTRACTION STUDIES OF SELECTED ACTINIDE IONS FROM AQUEOUS SOLUTIONS WTTH 4.BENZOYL-24-DIHYDRO-5-METHYL-2-PHENYL-3H-PYRAZOL-3-THIONE AND TRI-n-OCTYLPHOSPHTNE OXIDE. *Solvent Extr. Ion Exch.* **1992,** *10*, 431-438.

21. ALY, H. F.; LATIMER, R. M., Extraction of Transplutonium Elements by Di-2-Ethylhexylphosphoric Acid. *Radiochim. Acta* **1970,** *14*, 27-31.

22. Weaver, B.; Kappelmann, F. A., Preferential extraction of lanthanides over trivalent actinides by monoacidic organophosphates from carboxylic acids and from mixtures of carboxylic and aminopolyacetic acids. *J. inorg. nucl. chem.* **1968,** *30*, 263-272.

23. Kosyakov, V. N.; Yerin, E. A., Separation of transplutonium and rare-earth elements by extraction with HDEHP from DTPA solutions. *Journal of Radioanalytical Chemistry* **1978,** *43*, 37-51.

24. Thompson, M. C. *Distribution of selected lanthanides and actinides between 30% TBP in n-paraffin and various metal nitrate solutions*; United States, 01, 1973.

25. Watanabe, K.; Ono, R., The Extraction of Curium and Americium by Tri-n-Octyl Phosphine Oxide. *J. Nucl. Med. Technol.* **1964,** *1*, 75-76.

26. Gharibyan, N.; Dailey, A.; McLain, D. R.; Bond, E. M.; Moody, W. A.; Happel, S.; Sudowe, R., Extraction Behavior of Americium and Curium on Selected Extraction Chromatography Resins from Pure Acidic Matrices. *Solvent Extr. Ion Exch.* **2014,** *32*, 391-407.

27. Madic, C, Testard, F, Hudson, M J, Liljenzin, J O, Christiansen, B, Ferrando, M, Facchini, A, Geist, A, Modolo, G, Gonzalez-Espartero, A, and Mendoza, J de. Partnew - New solvent extraction processes for minor actinides - final report; Partnew - Nouveaux procedes d'extraction par solvant pour les actinides mineurs - rapport final. France: N. p., 2004. Web.

28. Marie, C.; Kaufholz, P.; Vanel, V.; Duchesne, M.-T.; Russello, E.; Faroldi, F.; Baldini, L.; Casnati, A.; Wilden, A.; Modolo, G.; Miguirditchian, M., Development of a Selective Americium Separation Process Using H4TPAEN as Water-Soluble Stripping Agent. *Solvent Extr. Ion*

Exch. **2019,** *37*, 313-327.

29. Suzuki, H.; Tsubata, Y.; Kurosawa, T.; Sagawa, H.; Matsumura, T., Continuous extraction and separation of Am(III) and Cm(III) using a highly practical diamide amine extractant. *J. Nucl. Med. Technol.* **2017,** *54*, 1163-1167.

30. Alyapyshev, M.; Babain, V.; Tkachenko, L.; Kenf, E.; Voronaev, I.; Dar'in, D.; Matveev, P.; Petrov, V.; Kalmykov, S.; Ustynyuk, Y., Extraction of actinides with heterocyclic dicarboxamides. *J. Radioanal. Nucl. Chem.* **2018,** *316*, 419-428.

31. Matveev, P. I.; Borisova, N. E.; Andreadi, N. G.; Zakirova, G. G.; Petrov, V. G.; Belova, E. V.; Kalmykov, S. N.; Myasoedov, B. F., A first phosphine oxide-based extractant with high Am/Cm selectivity. *Dalton Trans.* **2019,** *48*, 2554-2559.

32. Afsar, A.; Harwood, L. M.; Hudson, M. J.; Westwood, J.; Geist, A., Effective separation of the actinides Am(iii) and Cm(iii) by electronic modulation of bis-(1,2,4-triazin-3-yl)phenanthrolines. *Chem. Commun.* **2015,** *51*, 5860-5863.

33. Sasaki, Y.; Tsubata, Y.; Kitatsuji, Y.; Sugo, Y.; Shirasu, N.; Morita, Y., Novel Extractant, NTAamide, and its Combination with TEDGA for Mutual Separation of Am/Cm/Ln. *Solvent Extr. Ion Exch.* **2014,** *32*, 179-188.

34. Sasaki, Y.; Tsubata, Y.; Kitatsuji, Y.; Sugo, Y.; Shirasu, N.; Morita, Y.; Kimura, T., Extraction Behavior of Metal Ions by TODGA, DOODA, MIDOA, and NTAamide Extractants from HNO3 to n-Dodecane. *Solvent Extr. Ion Exch.* **2013,** *31*, 401-415.

35. Chapron, S.; Marie, C.; Arrachart, G.; Miguirditchian, M.; Pellet-Rostaing, S., New Insight into the Americium/Curium Separation by Solvent Extraction using Diglycolamides. *Solvent Extr. Ion Exch.* **2015,** *33*, 236-248.

36. Gujar, R. B.; Ansari, S. A.; Mohapatra, P. K.; Leoncini, A.; Verboom, W., Solid phase extraction of Am(III) and Cm(III) from acidic feeds using tetraethyl diglycolamide (TEDGA) in ionic liquid. *J. Radioanal. Nucl. Chem.* **2016,** *309*, 819-825.

37. Lange, S.; Wilden, A.; Modolo, G.; Sadowski, F.; Gerdes, M.; Bosbach, D., Direct Selective Extraction of Trivalent Americium from PUREX Raffinate Using a Combination of CyMe4BTPhen and TEDGA—A Feasibility Study. *Solvent Extr. Ion Exch.* **2017,** *35*, 161-173.

38. Zsabka, P.; Hecke, K. V.; Wilden, A.; Modolo, G.; Verwerft, M.; Binnemans, K.; Cardinaels, T., Selective Extraction of Americium from Curium and the Lanthanides by the Lipophilic Ligand CyMe4BTPhen Dissolved in Aliquat-336 Nitrate Ionic Liquid. *Solvent Extr. Ion Exch.* **2020,** *38*, 194-211.

39. Geist, A.; Müllich, U.; Magnusson, D.; Kaden, P.; Modolo, G.; Wilden, A.; Zevaco, T., Actinide(III)/Lanthanide(III) Separation Via Selective Aqueous Complexation of Actinides(III) using a Hydrophilic 2,6-Bis(1,2,4-Triazin-3-Yl)-Pyridine in Nitric Acid. *Solvent Extr. Ion Exch.* **2012,** *30*, 433-444.

40. Wagner, C.; Müllich, U.; Geist, A.; Panak, P. J., Selective Extraction of Am(III) from PUREX Raffinate: The AmSel System. *Solvent Extr. Ion Exch.* **2016,** *34*, 103-113.

41. Kaufholz, P.; Modolo, G.; Wilden, A.; Sadowski, F.; Bosbach, D.; Wagner, C.; Geist, A.; Panak, P. J.; Lewis, F. W.; Harwood, L. M., Solvent Extraction and Fluorescence

Spectroscopic Investigation of the Selective Am(III) Complexation with TS-BTPhen. *Solvent Extr. Ion Exch.* **2016,** *34*, 126-140.

42. Jensen, M. P.; Chiarizia, R.; Shkrob, I. A.; Ulicki, J. S.; Spindler, B. D.; Murphy, D. J.; Hossain, M.; Roca-Sabio, A. n.; Platas-Iglesias, C.; de Blas, A. s.; Rodríguez-Blas, T., Aqueous Complexes for Efficient Size-based Separation of Americium from Curium. *Inorg. Chem.* **2014,** *53*, 6003-6012.

43. Borrini, J.; Favre-Reguillon, A.; Lemaire, M.; Gracia, S.; Arrachart, G.; Bernier, G.; Hérès, X.; Hill, C.; Pellet-Rostaing, S., Water Soluble PDCA Derivatives for Selective Ln(III)/An(III) and Am(III)/Cm(III) Separation. *Solvent Extr. Ion Exch.* **2015,** *33*, 224-235.

44. Baldwin, A. G.; Ivanov, A. S.; Williams, N. J.; Ellis, R. J.; Moyer, B. A.; Bryantsev, V. S.; Shafer, J. C., Outer-Sphere Water Clusters Tune the Lanthanide Selectivity of Diglycolamides. *ACS Cent. Sci.* **2018,** *4*, 739-747.

45. Brigham, D. M.; Ivanov, A. S.; Moyer, B. A.; Delmau, L. H.; Bryantsev, V. S.; Ellis, R. J., Trefoil-Shaped Outer-Sphere Ion Clusters Mediate Lanthanide(III) Ion Transport with Diglycolamide Ligands. *J. Am. Chem. Soc.* **2017,** *139*, 17350-17358.

46. Jianchen, W.; Chongli, S., HOT TEST OF TRIALKYL PHOSPHINE OXIDE (TRPO) FOR REMOVING ACTINIDES FROM HIGHLY SALINE HIGH-LEVEL LIQUID WASTE (HLLW). *Solvent Extr. Ion Exch.* **2001,** *19*, 231-242.

47. Liu, L.-K.; Xie, S.-B.; Lv, H.-B.; Zhang, H.; Ye, G.-A., Studies on the separation treatment of high-level liquid waste by bisamide podand(I): Extraction and separation of An(III) from Ln(III). *Chin. Chem. Lett.* **2022,** *33*, 3439-3443.
